# Advances in natural product discovery: strategies, technologies, and insights

**DOI:** 10.1007/s13659-025-00556-1

**Published:** 2026-01-07

**Authors:** Buddha Bahadur Basnet, Zhen-Yi Zhou, Rajesh Basnet, Bin Wei, Hong Wang

**Affiliations:** 1 College of Pharmaceutical Sciences, Zhejiang University of Technology, Hangzhou, 310014 China; 2 Central Department of Biotechnology, Tribhuvan University, Kathmandu, Nepal; 3 Key Laboratory of Marine Fishery Resources Exploitment, Utilization of Zhejiang Province, Zhejiang University of Technology, Hangzhou, 310014 China; 4 CAS Key Laboratory of Regenerative Biology, Guangdong Provincial Key Laboratory of Stem Cell and Regenerative Medicine, Guangzhou Institutes of Biomedicine and Health, Chinese Academy of Sciences, Guangzhou, 510530 China; 5 University of Chinese Academy of Sciences International College, 19 Yuquan Road, Shijingshan District, Beijing, 100049 China

**Keywords:** Natural products, Culturing modulation, Unexplored reservoirs, Genome mining, Natural product diversification

## Abstract

Natural products (NPs) and their analogues have long underpinned therapies in humans, animals, and plants health, yet, discovering truly novel scaffolds remains a formidable challenge, even with the enormous diversity offered. Over the last two decades, breakthroughs in bioinformatics, cheminformatics, advanced analytical methods, synthetic biology toolkits, and optimized microbial culture have surmounted many of the bottlenecks that stalled NP research in the 1990s and 2000s. Researchers now deploy innovative extraction and purification protocols alongside high-throughput dereplication tools to fish trace metabolites out of complex matrices. These combined approaches not only enable the discovery and rigorous characterization of biosynthesized metabolites, bio-transformed analogues and new chemical entities but also allow precise tuning of biosynthetic gene clusters (BGCs) and culture conditions- modulation and optimization, dramatically improving yield, scalability, and cost-efficiency. Several of these newly unearthed compounds exhibit unique bioactivities that directly inspire drug-development programs against metabolic disorders, cancer drug resistance, and infectious diseases. In this review, we present an up-to-date, concise roadmap of natural product discovery (NPD), majorly covering strategies for awakening silent BGCs, genome mining, and late-stage diversification systems, and we discuss the current limitations and perspectives of rational NPD.

**Graphical Abstract:**

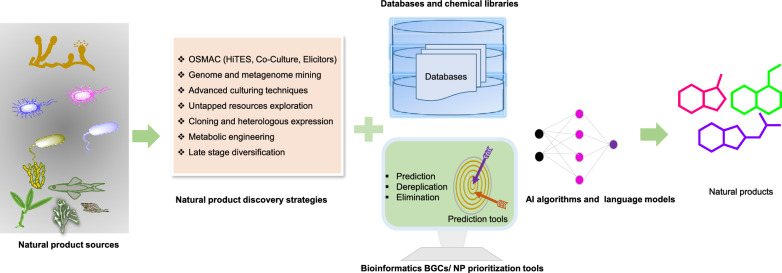

## Introduction

Natural products (NPs) are small organic molecules such as peptides, polyketides, saccharides, terpenes, and alkaloids, produced by plants, microbes, invertebrates and animals for self-defense or as metabolic byproducts. While non-essential for growth, NPs play a crucial role in chemical ecology, from defense against predators and competitors to sensing environmental cues like light [1–3]. Their biosynthesis proceeds via enzyme cascades and precursors from nutrient sources or primary metabolic pools, especially amino acids and tricarboxylic acid cycle intermediates, to assemble structurally diverse compounds [2, 4].

NPs and their semi-synthetic analogs form a rich reservoir of pharmacologically potent compounds whose structural diversity underlies a broad spectrum of bioactivities, from antimicrobial and antiparasitic to anticancer effects. This chemical versatility has driven drug discovery for millennia, delivering new therapeutic leads long before modern screening platforms existed. That legacy endures today with over half of the US Food and Drug Administration-approved drugs from 1939 to 2019 derived from NPs or their derivatives [5].

Tracing back to history, “morphine” from *Principium somniferum* and the semi-synthetic drug "aspirin" based on salicin, an NP from Salix, being the first proof as a pure and NPs derivative practiced for human disease treatment [6, 7] Over the eons, classical trial-and-error top-down approaches, such as structure-, bioactivity, and affinity- guided bioactive molecules isolation [8], contributed to significant early milestones in drug approval. For instance, 70–80% of antibiotics discovered were directly NPs based or inspired by NPs entities. The period from 1940 to 1960s is often regarded as the “golden age” in natural products drug discovery history [9, 10]. During this time, intense research into microbial sources, especially soil-dwelling bacteria like *Streptomyces*, led to the rapid identification of numerous antibiotic classes [11], antipsychotic agents [12], and anticancer agents [13] that are still in clinical use today.

**Fig. 1:**
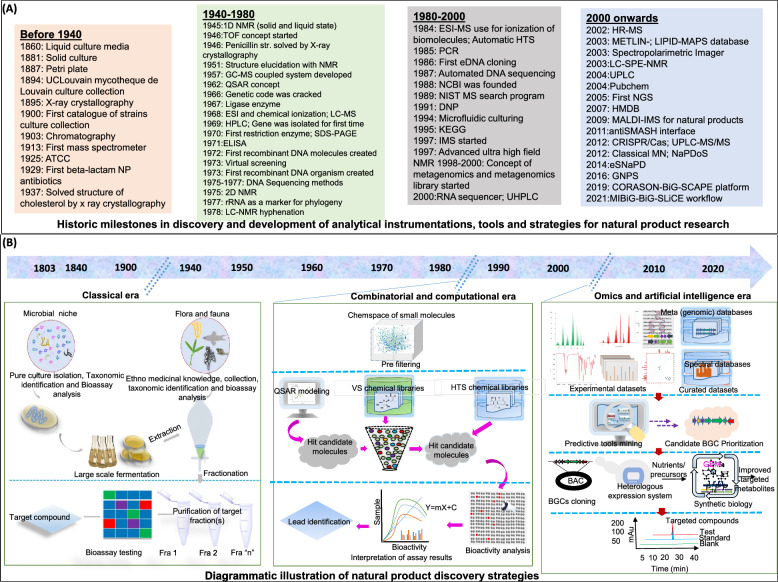
Progress time trend in hallmark tools and strategies workflow for accelerating natural product discovery (NPD). **A** Significant discoveries time of instruments, databases and dereplication tools in NPD research. **B** Time trend in isolation techniques majorly, ancient to late-twentieth century period (Top-Down Approach), late 20th to early 21th century period (Virtual Screening, High Throughput Screening and Combinatorial-/biosynthesis) and modern early 21th century to now (Omics, Artificial Intelligence and Bottom-Up Approach)

**Fig. 2:**
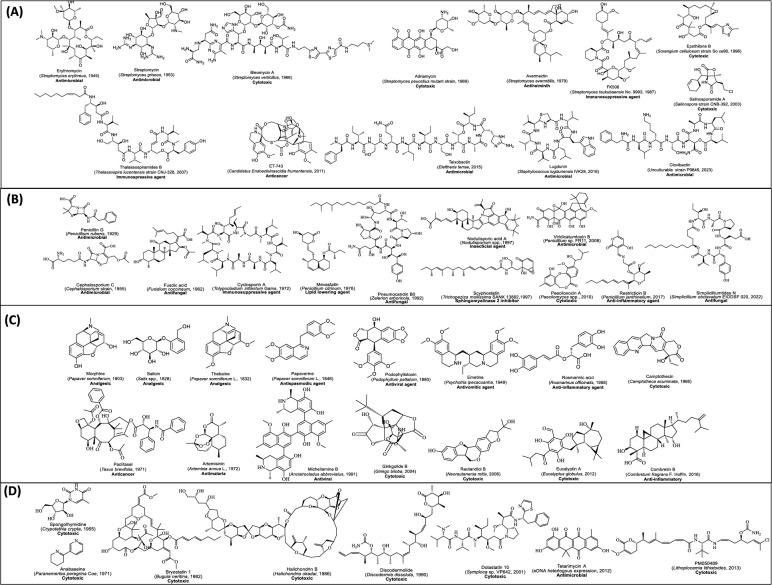
Representative revolutionary natural product discovered during the 1800–2023, usually in every decade. **A** Natural products (NPs) isolated from bacterial source, **B** NPs isolated from fungal source, **C** NPs isolated from plant source and **D** NPs from miscellaneous sources. Each metabolite is listed with its name, source of first-time isolation (provided in parentheses), and biological activity (highlighted in bold)

Figure 1 illustrates the chronological progression of essential tools, methodologies, and landmark discoveries that have propelled natural product discovery (NPD) from 1800 to 2024. It highlights major technological milestones, such as instruments, databases, and dereplication tools, alongside key isolation strategies exploited maximum during that period. Figure 2 displays notable NPs discovered across this timeline, emphasizing their origins from microbial and plant sources and detailing their biological activities.

Ethno medicine, phenotypic screening and bioactivity-guided trial-and-error methods have traditionally served as widely adopted protocols for NPs identification and discovery in the classical era. However, these trial-and-error methods constraints such as poor cultivability under standard culture settings, high rediscovery rates, labor and time-intensive, and unnecessary financial burden diminished the natural products scientists stake and pharmaceutical industries on NPs research [14]. In this scenario, high throughput screening (HTS) and combinatorial synthesis intensively exploited between the 1980s to early 2000s improved the lead discovery by increasing the hit rate to approximately 10–40% [15], but these systems have their own set of restrictions, such as limited chemical diversity and screening library chemspace size [16]. Therefore, they failed to meet continuous demand for new chemical entities (NCEs), new scaffolds and drugs as highlighted by a survey reporting only two Food and Drug Administration-approved combination drugs over a 39-year period [5].

Amid this backdrop, cutting-edge "bottom-up approaches" including multi-omics technologies, hyphenated analytical techniques, and bio-/cheminformatics platforms, offer exciting avenues for accelerating the novel carbon skeletons or NCEs discovery. Coupled with advanced mass spectrometry (MS)/ nuclear magnetic resonance (NMR)-based dereplication methodologies and databases (Table 1), which swiftly filter known compounds and eliminate redundancy, these innovations enable precise targeted exploration of unique metabolites [17]. This era is often regarded as a “new golden period” or “muti-omics AI era” for NPs-based drug discovery and development [18]. Concomitantly, the rapid surge of NPs, with approximately 6–7 million standardized and centralized in public databases such as Dictionary of Natural Products, COlleCtion of Open Natural prodUcTs, Natural Product Atlas (Table 1A**)**, accounts for only 1/10th of the total NPs chemspace [2]. Yet, specialized metabolites produced in low concentrations (often less than 1% by weight) in complicated cellular compartments, a time-consuming dereplication process, silent or incomplete biosynthetic domains nature of biosynthetic gene clusters (BGCs) remain major challenges [19].

**Table 1:** Databases and mining tools in natural product discovery

Database/tools	Description	Website or Github link	References
*A. Most curated natural products (NPs) database*			
DNP (Version 32.2)	~ 340,000 NPs from diverse resources	http://dnp.chemnetbase.com/	
CMNPD	~ 31 000 marine NPs	https://cmnpd.org/	[20]
COCONUTS	~ 730,441 NPs	https://coconut.naturalproducts.net/	[21]
NP Atlas	~ 249 594 compounds	www.npatlas.org	[22]
LOTUS	~ 276 518 NPs	https://lotus.naturalproducts.net	[23]
Super natural II DNP	~ 325 000 NPs	http://bioinformatics.charite.de/supernatural	[24]
NPASS	~ 94 413 NPs with activity ~ 43 285 without activity ~ 51 128	http://bidd.group/NPASS	[25]
HMDB (version 5.0)	~ 217 920 compounds	https://hmdb.ca	[26]
ZINC-NP	~ 150 000 NPs	http://zinc.docking.org/	[27]
*B. Hidden Markov Model (HMM) based BGCs annotation tools*			
SMURF	Predicts clustered SM genes based on genomic/domain content	www.jcvi.org/smurf/	[28]
Anti-Smash (Version 7)	Detects and characterizes BGCs in genomes	https://antismash.secondarymetabolites.org/	[29]
CLUSEAN	BLAST and HMMer integration for annotating gene clusters	https://bitbucket.org/tilmweber/clusean	[30]
ClustScan	Annotates modular BGCs and predicts chemical structures		[31]
Cluster Finder	Predict BGCs in genomes using heuristic approach	https://github.com/petercim/ClusterFinder	[32]
np.searcher	Predicts SMILES of polyketide and NRPS using DNA input	https://dna.sherman.lsi.umich.edu/	[33]
SeMPI version 2.0	Pipeline for predicting polyketides and NRPS	sempi.pharmazie.uni-freiburg.de	[34]
SBSPKS	Predicts PKS catalytic domains and substrate specificity	http://www.nii.ac.in/sbspks.html	[35]
*C. AI integrated genome mining dereplication tools*			
antiSMASH, MIBiG, Big-SCAPE & CoRASON platform	Provide identification, compare and correlate the biosynthetic information with secondary metabolites in databases, and link evolutionary and maps phylogenetically	https://bigscape-corason.secondarymetabolites.org, https://git.wur.nl/medema-group/BiG-SCAPEhttps://github.com/nselem/corason	[36]
RiPPQuest, NRPQuest, Pep2Path, NRPSPredictor2 and NPOmix	Molecular networking approach identify the potential gene clusters or gene cluster families from analytical datasets (e.g. MS/MS fragmentation dataset)		[37, 38]
BiG-SCAPE & CORASON together with BiG-SLICE workflow	Enable reconstruction of BGCs phylogenies from different sources and groups into gene cluster families	https://github.com/medema-group/bigslice	[39]
RODEO, GECCO, RiPPER & RiPPMiner	Identify unique superclusters or multi-precursor peptide RiPP BGCs based on special enzyme features guided by phylogeny closeness, or by chemocentric searches	https://github.com/streptomyces/ripper http://www.ripprodeo.org	[40, 41]
DeepRiPP, DeepBGC, SanntiS, NeuRiPP, decRiPPter & RRE-Finder	Deep learning neural approaches that used multiomics data to automate discovery of novel ribosomally synthesized NPs	http://rodeo.scs.illinois.edu https://github.com/Alexamk/RREFinder https://github.com/Merck/deepbgc	[41, 42]
BAGEL4, SANDPUMA, GNP, iSNAP & MetaMiner	Feature-based tools that predicting specific metabolite classes such as bacteriocin, nonribosomal peptides, polyketides, and ribosomally synthesized and post-translationally modified peptides	http://bagel4.molgenrug.nl/	[40, 41]
BiG-FAM database	A user-friendly interface to facilitate the display and comparison of gene clusters directly from query sequences	https://bigfam.bioinformatics.nl	[43]
PRISM-4, DDAP, AdenPredictor, & PKSpop	Predict chemical structure from genome sequences		[44]
NPlinker, EFI-CGFP, MAGI	Scoring functions and pattern-recognition integrated approaches for identifying new chemcial entities, metabolic pathways, or BGCs by recognizing unique features from the BGCs, novel enzyme encoding BGCs domains and biochemical predictions for poorly annotated genes		[44]
*D. Metabolic pathway databases*			
KEGG (Version 80.2)	~ 4000 complete genomes annotated with KOs in KEGG database 16 databases grouped into 4 categories: systems, genomic, chemical, and health information 10,307 reactions 17,787 compounds 474,838 pathways 6836 proteins	http://www.kegg.jp/	[45] [46] [47]
ModelSEED	33 978 compounds 36 645 reactions 28 120 structures Contains reactions from KEGG, MetaCyc, BiGG, MetaNetX and Rhea	http://modelseed.org	[48]
Rhea	14,583 unique reactions 12,601 unique reactants	https://www.rhea-db.org	[49]
BiGCARP	Capture meaningful patterns in BCGs with AUROC score from 0.936—0.950	https://github.com/microsoft/bigcarp	[50]
BioCyc	~ 20 025 pathway/genome databases for model eukaryotes & microbes	http://biocyc.org	[51]
BiGG	Contains 108 curated GEMs for prokaryotes and eukaryotes, with standardized metabolite and reaction identifiers	http://bigg.ucsd.edu	
Brenda	Provides information for ~ 8400 enzymes Data stored in about 50 categories	www.brenda-enzymes.org	[52]
EcoCyc (version 26.1)	Contains more than 20 000 microbes informations 4546 genes 41,346 protein features 2202 metabolic reactions 3694 transcription units	https://ecocyc.org	[53]
MetRxn	~ 76,000 metabolites 72,000 reactions	http://metrxn.che.psu.edu	[54]
MetaCyc (version 27.1)	3128 pathways 18,819 reactions 14,320 enzymes 1973 chemicals	MetaCyc.org	[55]
*E. Metabolic pathway construction tools*			
MetaDraft	Create Genome-scale Metabolic Model (GSMMS) from manually curated models, use BIGG templates	https://systemsbioinformatics.github.io/cbmpy-metadraft/	[56]
Merlin	Java application for genome-scale reconstruction based on KEGG database	https://merlin-sysbio.org/	[57]
MRE	Designs and optimizes metabolic pathways	http://www.cbrc.kaust.edu.sa/mre/	[58]
BlastKOALA	Analyzes genes and genomes, performs functional characterization Pathway analysis and metagenome analysis	http://www.kegg.jp/blastkoala/	[59]
GhostKOALA	Comprehensive analysis of genes from metagenomes	www.kegg.jp/ghostkoala/	[59]
RAST	Annotates bacterial and archaeal genomes, identifies protein-coding genes, assigns functions	http://rast.nmpdr.org	[60]
KAAS	Automatic genome annotation and pathway reconstruction	http://www.genome.jp/kegg/kaas/	[61]
AuReMe	Reconstructs microorganism models, creates GSMMs with a template-based algorithm	https://aureme.genouest.org/	[57]
CarveMe	Command-line tool for creating GSMMs, ready for flux balance analysis	https://github.com/cdanielmachado/carveme	[57]
RAVEN (Version 2.0)	Semi-automatic toolbox for reconstruction, curation, and simulation of metabolic models	(https://github.com/SysBioChalmers/RAVEN)	[62]
Pathway tools (Version 23.0)	Manages and analyzes organism-specific database called Pathway/Genome Databases (PGDBs)	https://bioinformatics.ai.sri.com/ptools/	[63]
*F. Resistance gene mining bioinformatics tools and databases*			
ARTS-DB	Repository with > 70,000 genome results for genome mining, prioritizing BGCs for novel antibiotics	https://arts-db.ziemertlab.com/	[64]
CARD	Contains 322,710 unique ARG allele sequences, well-characterized resistance genes, their products, and a bait capture platform	https://card.mcmaster.ca/	[65]
DeepARG	Uses deep learning to annotate antibiotic resistance genes in metagenomes	https://bench.cs.vt.edu/deeparg	[66]
ARG-ANNOT	Detects antibiotic resistance genes in bacterial genomes using local BLAST in Bio-edit software, without a web interface	https://www.mediterranee-infection.com/acces-ressources/base-de-donnees/arg-annot-2/	[67]
FunARTS	Links housekeeping and resistant genes to BGCs for automated, site-directed mining of fungal genomes	https://funarts.ziemertlab.com	[68]
FRIGG	Identify paralog of the target resistance gene in biosynthetic gene cluster based on homology patterns of the cluster genes		[69]
BacMet	Identifies biocide and metal-resistance genes in full genomes. Version 2.0 has 753 confirmed and 155,512 predicted resistance genes	http://bacmet.biomedicine.gu.se/	[70]
RGDB	Resistance gene compiled from CARD, MIBiG, NCBIAMR, and Uniprot		[71]
ResFinder	Used for identification of antimicrobial resistance genes in next-generation sequencing data and prediction of phenotypes from genotypes	https://cge.cbs.dtu.dk/services/ResFinder/	[72]
GraphAMR	ARG detection in Complex Metagenomics Datasets	https://github.com/ablab/graphamr	[73]
ResFams	Identifies protein families linked to antibiotic resistance, including acetyltransferases, Arac transcriptional regulators, MFS transporters, ABC transporters, and efflux pumps	https://www.dantaslab.org/resfams	[74]
MEGARes	Contains antimicrobial resistance, metal and biocide resistance determinants sequences	https://megares.meglab.org	[75]

Additionally, modern bioinformatics analysis, metagenomics and next-generation sequencing illuminated that the number of BGCs is considerably underappreciated compared to expected [76], highlighting the tremendous potential for NCEs from untapped biodiversity [77]. Further, profound improvement in mass spectrometry studies disclosed that the number of secreted NPs far surpasses the number of BGCs, which paved the way for a logic for myriad unconventional NPs. Given the limitations of classical approaches, recent advancements in metabologenomics, next-generation synthetic biology, and cutting-edge technologies such as artificial intelligence (AI), machine learning (ML), and large language models have significantly improved both the hit rate and the yield of structurally diverse carbon skeletons and their analogues. Moreover, emphasis has been placed on combinatorial approaches [37], particularly with the expansion of virtual screening libraries to ultra-large scales [78] and the implementation of platforms such as VirtualFlow [79]. These strategies are often coupled with ultrasensitive automation, substantial miniaturization, or whole-cell phenotypic high-throughput screening (HTS) techniques [80], leveraging massive datasets like the L1000 [81] to enhance screening efficiency. Nevertheless, robust high-throughput methodologies for the comprehensive detection, isolation, and characterization of all encoded natural products and their full chemical diversity from complex extracts remain elusive.

NPs offer tremendous promise for developing novel therapeutics and advancing sustainability in food and agriculture. However, despite their vast potential, technical, biological, and regulatory hurdles continue to constrain their discovery and translation into practical applications. NP discovery generally begins with metabolite isolation via chromatography and bioassay-guided fractionation, followed by structural elucidation using advanced spectroscopic methods such as MS, NMR, chemical derivatization, and X-ray crystallography. Although many NPs exhibit potent bioactivity, issues like poor solubility, chemical instability, and toxicity can impede pharmacological development. Conversely, their ability to interact with multiple biological targets opens opportunities for multitarget therapies while also raising concerns about off-target effects. Early dereplication helps identify known compounds and reduce redundancy, yet it underscores the difficulty of finding truly novel entities. To overcome these challenges, advances in analytical instrumentation and informatics, especially AI-driven platforms and deep-learning tools in bioinformatics and cheminformatics, combined with next-generation synthetic biology now enable high-confidence prediction, reconstruction, and expression of BGCs and metabolic pathways. Emerging and updated tools and strategies such as antiSMASH, Global Natural Products Social Molecular Networking (GNPS), High Throughput Elicitors Screening (HiTES), resistance/phylogeny-guided genome mining, transcriptional/translational modulation, and late-stage modification are becoming indispensable components of the modern NP discovery pipeline.

## Important pillars of natural product discovery: osmac, co-culture, and elicitors

Classical methods like bioassay-guided fractionation, top-down approaches, and structure elucidation via NMR, X-ray, and MS, alongside culture optimization techniques like one strain many compounds (OSMAC) [82] and HiTES [83], continue to thrive in unveiling NCEs maintaining its effectiveness even amid the remarkable success of modern discovery approaches.

### OSMAC

**Fig. 3:**
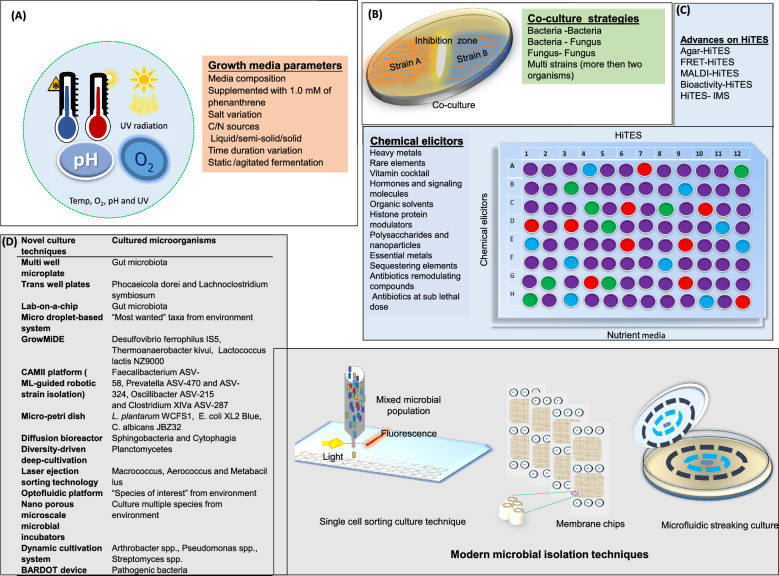
Culture modulating and untapping the unexplored reservoirs techniques. **A** Physical and chemical methods **B** Co-culture strategies. **C** HiTES experiment. **D** Examples of modern microbial cultivation techniques

OSMAC is a modified culturing technique widely used in NP research to expand chemical diversity and trigger cryptic gene expression for cryptic specialized metabolites by systemic altering environmental and chemical triggers like media composition, carbon sources, and fermentation methods and listed in Fig. 3A. Despite its low-tech simplicity, this approach has proven remarkably powerful across diverse sources, including marine resources [84], symbiotic fungi [85], lichen [86], and *Streptomyces* [87] to discover plethora of novel NPs. For example, recent comprehensive reviews by Zhang et al*.* [88] and *Zhu and *Zhang [89] reported the discovery of over 284 microbial cyclic peptides from 63 endophytic strains, and 476 secondary metabolites from fungal strains, respectively, through the application of the OSMAC strategy, illuminating the strategy’s huge potential for NPs discovery.

One major limitation of this approach is the prioritization of compounds within a complex crude extract, which contains a vast chemical space. This complexity poses significant challenges for subsequent steps such as compound isolation and purification. In recent years, efforts to simplify the dereplication process have involved OSMAC-modified strategies, including integration with heterologous expression systems [90], molecular networking [91], genome mining [92], metabolic shunting [93] to speculate optimum conditions for discovering novel NPs, or analogues. For instance, Esposito et al*.* [92] isolated more than 30 new glycolipids with unusual functional groups from the marine bacterium *Rhodococcus* sp. I2R by combining an OSMAC approach with genome mining and advanced metabolomics analysis. Similarly, Wu et al. [94] applied genome mining alongside an OSMAC strategy to uncover 12 new alkaloids from termite-associated *Streptomyces tanashiensis* BYF-112.

### Co-culture

Co-culture techniques leverage interactions between multiple microorganisms, either on solid or in liquid media, to enhance NPD. By mimicking ecological niches and chemical crosstalk, co-culture can activate silent biosynthetic gene clusters or trigger the scalable production of specialized metabolites via poorly understood signals [95], except with little evidence of the mimicry of ecological culture niches and interspecies/intraspecies crosstalk [96]. Unlike monoculture, which often silences key BGCs and requires complex genetic or bioprocess interventions, co-culture offers a simple promising alternative for activating and enhancing the yield of diverse NPs [97]. For example, Peng et al. [98] and Li et al. [97] reported the discovery of 93 novel bioactive natural products from co-cultured microorganisms (2017–2020) and 69 metabolites from various co-culture techniques, respectively.

Figure 3B illustrate possible combination of co-partner in co-culture strategies. Moreover, basic setups include mixed growth, spatial separation via layered or immobilized arrangements, and encapsulation in shared media to limit dominance. In other designs, partners are placed in distinct chambers that exchange metabolites through semi-permeable membranes or volatile signals across a gas interface. Alternatively, one strain spent media or extract can be used to stimulate the metabolic activity of the other [97].

Recent technological advances have further empowered co-culture. Biosensor-assisted cell selection strategy [99], optogenetics circuit system [100], microbe-laden hydrogel system [101] and metabolic flux analysis [101] allow real-time monitoring and dynamic control of species interactions. For instance, Guo et al*.* [99] engineered two separate *Escherichia coli–Escherichia coli* co-culture systems, one channeling 4-hydroxybenzoate into phenol, the other tyrosine, each equipped with a phenol biosensor incorporating a biosensor to monitor phenol production, which resulted in 2.3- and 3.9-fold increases in phenol titers, respectively.

Furthermore, integration with advanced analytics, for instance, combined with imaging mass spectrometry techniques such as MALDI-TOF–MS and Nano-DESI-IMS [102], as well as high-throughput elicitor screens (HiTES) [103], have significantly uncovered target NPs and their derivatives. In one study, coupling a bioassay with HiTES in a co-culture system led by Moon et al. [103] to isolate the novel lanthipeptide antibiotic cebulantin.

More recently, a new approach termed “modular co-culture engineering” has been introduced to address the shortcomings of monoculture fermentation and improve the enhance NPs biosynthesis. In this strategy, a complex biosynthetic pathway into distinct functional modules, each assigned to a different microbial strain. Each strain is independently engineered and optimized prior to co-culturing (either together in shared media or within compartmentalized systems), to produce the target compound. By balancing population ratios and monitoring metabolite exchange, this approach reduces metabolic stress on individual microbes and improves overall biosynthetic efficiency [104, 105]. For example, applying this strategy, Marsafari et al. [106] co-cultured engineered *Yarrowia lipolytica* Po1f and Po1g strains, and reported an increase of amorphadiene titer of 60–70 mg/mL, compared to 40 mg/mL in monoculture.

### HiTES

HiTES is an innovative technique designed to unlock the hidden biosynthetic potential of microorganisms by activating silent or cryptic BGCs with libraries of small-molecule elicitors. Unlike traditional genetic manipulation methods, HiTES operates without the need for cloning or genome editing, making it a rapid and versatile platform [83] (Fig. 3C). However, no established methodology currently exists for identifying small-molecule elicitors that selectively activate a specific silent BGC. This shortfall has slowed efforts to unravel the complex regulatory networks controlling secondary metabolite expression and left many potentially valuable natural products dormant. Reporter constructs offer a partial solution by linking induction of a target BGC to an easily measurable signal. In one early demonstration, inserted a reporter into multiple gene clusters and screened small-molecule libraries, identifying sub lethal trimethoprim as a global inducer of at least five BGCs [107]. Building on this, *Xu *et al. (2017) engineered an eGFP reporter into the sur BGC, which led to the characterization of 14 novel surugamide-family metabolites following elicitor treatment [108].

Another major challenge in HiTES workflows is managing and interpreting the exceptionally complex LC–MS datasets that each elicitor screen produces. Automated dereplication against spectral libraries (e.g. GNPS) [109] and *in-silico* fragmentation tools (e.g. SIRIUS/CSI:FingerID) [110] help to annotate known compounds, but unknowns remain pervasive. GNPS, conceptually initiated in 2011 and introduced in 2014, is an open-access, web-based platform that facilitates the community-driven sharing and analysis of MS/MS data. It supports a variety of advanced workflows that enhance analytical resolution and throughput by integrating complementary modalities [111]. It have been widely applied across diverse sources including plants, microorganisms, and extremophiles for the discovery of all classes of NPs [112, 113]. GNPS advances include feature-based molecular networking (FBMN) which improves molecular comparisons by incorporating fragmentation spectra, isotope patterns, retention times, and ion mobility data [114]. Ion Identity Molecular Networking extends FBMN by linking ion species of the same molecule based on known mass differences, adding an MS^1^-level connectivity layer [115]. Building Blocks-Based Molecular Networking combines neutral loss scanning with molecular networking to identify biogenetically relevant metabolites and streamline MS^2^ datasets through feature filtering [116]. Substructure-Based Molecular Networking applies unsupervised learning to detect recurring molecular fragments, known as “Mass2Motifs,” across spectra [117, 118]. Bioactivity-Based Molecular Networking integrates chemometric analysis to distinguish active from inactive compounds in complex mixtures, although it does not provide structural details [119].

In addition, multivariate statistical analyses then correlate elicitor identity with metabolomics shifts, flagging high-priority “hits” for follow-up [120]. Recently, interactive visualization platforms such as MetEx, generates interactive multidimensional and 2D plots, enabling global metabolome visualization, cryptic metabolite prioritization, dereplication, elicitor structure–activity relationship analysis, and ranked lead selection [121].

In addition, advanced HiTES variants integrated analytical or imaging modalities boost throughput and resolution. MALDI-HiTES couples MALDI-TOF mass spectrometry with HiTES for rapid metabolite prioritization [122]. Bioactivity-HiTES incorporates assay workflows for direct activity screening [123], HiTES-IMS leverages imaging mass spectrometry [124], and FRET-HiTES uses fluorescent resonance energy transfer sensors to report induction events [125]. For example, Zhang and Seyedsayamdost [122] applied MALDI-HiTES to *Streptomyces ghanaensis*, rapidly prioritizing and identifying the cryptic non-ribosomal peptide cinnapeptin.

### Chemical elicitors

In diverse ecological niches, microorganisms use NPs as chemical signals to communicate within and between species, coordinate resource use, and trigger metabolite production [126]. In the lab, a wide array of chemical elicitors, including rare elements, heavy metals, hormones, signaling molecules, sulfo-compounds, organic solvents, histone inhibitors, polysaccharides, nanoparticles, metal sequestering agents, and sub-lethal antibiotics, have shown to enhance diverse NP biosynthesis, improving NPD efficiency and yield [82]. Table 2 summarizes several proprietary chemical elicitors and the concentrations (millimolar to nanomolar) at which they activate or repress genes associated with silent BGCs such as metallosensor gene [127], although their precise molecular mechanisms often remain elusive [128]. Nonetheless, discovering new chemical elicitors and optimizing HTS from large libraries remain critical challenges for targeted BGC activation in microbial systems. To overcome this, many groups now combine computational prioritization with miniaturized bioassays (e.g. microplate or droplet-based) to triage hundreds to thousands of compounds in a single run. This integrated pipeline narrows down candidates by predicting which small molecules are most likely to bind regulatory elements or trigger reporter signals, and then validates hits in rapid fluorescence, mass-spec, or bioactivity readouts (see Sect. 2.3). For example, Han et al. [125] used a fluorescence-based DNA-cleavage assay on a 400-compound library and pinpointed five steroidal elicitors that rapidly induced cryptic enediyne production in *S. clavuligerus*.

**Table 2:** Chemical elicitors and selected natural products (sources)

Chemical elicitors	Concentration	Selected examples (sources)	References
Rare elements			
Scandium	100–500 μM	Actinorhodin (*Streptomyces coelicolor* A3(2))	[129]
	5–20 μM	Toyocamycin (*S. diastatochromogenes* SD3145)	[130]
Lanthanum	2 mM (1700–2500 μM)	Urauchimycin D (Actinobacter strain R818)	[131]
Heavy metals			
Cu^2+^, Zn^2+^, Cd^2+^, and Cr^3+^	0.5 mM CuSO_4_, 0.5 mM ZnSO_4_, 0.125 mM Cd(NO_3_)_2_, 0.0125 mM K_2_Cr_2_O_7_	Monocillin I (*Paraphaeosphaeria quadriseptata*)	[132]
Co^2+^ + Zn^2+^	0.5–4 mM	Anhydromevalonolactone (*Streptomyces* sp. SH-1312)	[133]
Mn^2+^	6 mM	Tacrolimus (*Streptomyces tsukubaensis*)	[134]
Ni^2+^	100 μM NiCl_2_·6H_2_O	Stremycin A and B (*Streptomyces pratensis* NA-ZhouS1)	[135]
Co^2+^	6 mM	*Neocitreoviridin, Penicillstressol, Isopenicillstressol (Penicillium* sp. BB1122)	[136]
Ni^2+^ and Fe^2+^	3.05 mM NiCl_2_ 1.33 g/L FeSO_4_	Melanin (*Streptomyces* sp. ZL-24)	[136]
Hormones and singaling molecules			
Salicylic acid	75 µM	Actinidine (*Nardostachys jatamansi)*	[137]
Methyl jasmonate	2 mM	Madecassic acid and asiatic acid (*Centella asiatica* (L.) Urban)	[138]
	75 µM	Glaziovine* (N. jatamansi)*	[137]
γ-butyrolactones	4 nM avenolide	Avermectin (*Streptomyces avermitilis*)	[139]
Sulfo-compounds and organic solvents			
DMSO	1–5%	Tetracenomycin C (*Streptomyces glaucescens*) Chloramphenicol (*Streptomyces venezuelae* strain ATCC1071) Thiostrepton (*Staphylococcus azureus* (ATCC14921))	[140]
Ethanol	6%	Jadomycin (*S. venezuelae* ATCC 10712)	[141]
	1–200 mM	Validamycin A (*Streptomyces hygroscopicus* 5008)	[142]
Hydrogen peroxide	25 μM	Validamycin A (*S. hygroscopicus* 5008)	[143]
Propionic acid	2–8 mM	Citric acid (*Aspergillus niger*)	[144]
Pyridine, imidazole, and methylheptenone		Lycopene (*Blakeslea trispora* and* Phycomyces blakesleeanus)*	[145]
Histone and other enzyme inhibitors			
Sodium butyrate	150 mM	Selvamicin (*Pseudonocardia* LS1)	[146]
	25 mM	Actinorhodin (*S. coelicolor* A3(2) strain M145)	[147]
Trichostatin A	1 μM	Cytochalasin E (*Aspergillus clavatus)*	[148]
Nicotinamide	50 μM	Chaetophenol G, cancrolides A and B (*Chaetomium cancroideum*)	[149]
5-azacytidine	0.1 μM–10 mM	Lunalides A and B (*Diatrype* spp.)	[150]
		Oxylipins (*Cladosporium cladosporioides*)	[150]
Phenobarbital	10–1000 μM	Ganoderic acids* (Ganoderma lucidum)*	[151]
Tricyclazole	5 ppm	Sphaerolone and dihydrospaerolone (*Sphaeropsidales* sp. F-24′707)	[152]
Suberanilohydroxamic acid	0.1 μM–10 mM	Perylenequinones (*C.cladosporioides*)	[153]
Polysaccharide and nanoparticles			
*N*-acetylglucosamine	0.5 μM	Fridamycins H and I (*Actinokineospora spheciospongiae* sp. Nov)	[154]
Chitosan	100–400 mg/L	Rosmarinic acid and quercetin* (Dracocephalum kotschyi)*	[155]
40-nm CuO nanoparticles		Actinorhodin (*S. coelicolor*)	[156]
Essential metal sequestering agents			
EDTA	10 mM	LNM K-3, verticilactam (Deep-Sea Bacteria)	[157]
2,2′-bipyridyl	350 μM	Actinorhodin (*S.coelicolor* A3(2) M145*)*	[158]
Antibiotics at sublethal dose and antibiotics remodeling compounds, ribosome-targeting drugs			
Triclosan	0.1–20 μM	Salinomycin (*Streptomyces albus*)	[159]
Ivermectin b1a	30 μM	Surugamides class of compounds (*S. albus* J1074)	[108]
Etoposide	23 μM	Surugamides class of compounds (*S. albus* J1074)	[108]
Monensin	7.54 μM	SF2768 (*Streptomyces griseorubiginosus* strain 574)	[160]
Antibiotics remodeling compounds	10 μM	Oxohygrolidin (*Streptomyces ghanaensis* ATCC 14672) 9- methylstreptimidone (*Streptomyces hygroscopicus* ATCC 53653)	[161]

Beyond chemical triggers, abiotic stress conditions like UV/vis irradiation and heat shock (Fig. 3A) also influence discovery and expansion of NP diversity [150]. Engineering the physical culture environment can mimic natural habitats: growing sponge-associated *Pseudoalteromonas* on cotton balls significantly increased levels of thiomarinol A, violacein, and bromo-alterochromide analogues [162], while adding inorganic talc microparticles accelerated morphological development in actinobacteria and improved oxygen diffusion in *Aspergillus terreus* mycelia, enhance lovastatin yields [163, 164].

All the approaches mentioned above are low-tech, straightforward methods used to activate silent BGCs or enhance the biosynthesis level to detectable range and are widely employed in NPs research laboratories. However, how to select the effective elicitors, growth parameters, and co-culture partners are central questions for all natural products scientists. Indeed, at present, selecting chemical elicitors or growth parameters or co culture partner(s) to activate BGCs involves a hit-and-trial. Despite their convenience and accessibility, these methods are always time-consuming and inefficient. This knowledge gap exemplifies the utmost need for the development of novel tools/strategies to predict appropriate elicitors or growth parameters for decoding the silent BGCs in vivo rather than complicated systematic trial and error approaches. Additionally, Table 3 outlines the advantages and disadvantages associated with each method.

**Table 3:** Highlights and limitations of natural product discovery strategies

Strategies	Advantage	Disadvantage
HiTES	Highly reproducibility Researcher direct control over variables Precise data collection and conclusion	Artificial conditions often fail to reflect real-world environments accurately High resource costs Influenced by researcher expertise, cultural context, and experimental parameters
OSMAC	Cost effective and easy to apply Enhances metabolite diversity using a single strain, avoiding new isolations Easily adaptable across media, temperature, and stress conditions	Strain genetics determine compound yield and metabolite diversity Scaling fermentation requires complex optimization from microliters to multiliters Optimization is time-consuming, and conditions may fail during scale-up
Co-culture	Mimic natural conditions to enhance known metabolites and produce new secondary metabolites Often activates silent genes, stimulating new metabolite discovery Cost effective and easy to apply	Growing different species can require a complex setup, making scale-up tough Multi-organism culture is complicated, making it difficult to identify species-specific metabolites Standardizing conditions and selecting compatible partners is tough in artificial cultures
Use of chemical elicitors	An efficient, cost-effective, and simple method for resources limited lab Induces or enhance the silent biosynthetic genes to uncover new metabolites Applicable to microorganisms, marine organisms, and plants	Identifying target elicitors and optimizing conditions simultaneously is challenging Certain elicitors can be toxic Elicitors effective in small-scale fermentation may not yield consistent results the in large-scale production
Advanced culturing techniques	Improves environmental control and enables high-throughput culturing Supports automation and scalability Enhance productivity and efficiency in valuable natural product production Enables natural product discovery from poor-to-culture or previously uncultured microorganisms	Inaccessible to all labs Require a complex setup and precise optimization Needs specialized equipment and expertise
Metagenomics approach	Covinent for uncultured microorganisms in their natural habitat Offers deeper insights into diverse DNA compositions and genetic variability High-throughput screening of bioactive natural products using functional assays	Expensive and requires expert analysis Not all predicted genes or BGCs express successfully in heterologous systems Functional gene linking is limited
Resistance gene mining	Highly effective for discovering new antimicrobial agents Provides deeper insight into the functional role of resistance Reduce the chance of rediscovery	Expensive and requires expert analysis Not all predicted resistance genes or BGCs express successfully in heterologous systems Not all predicted resistance genes are linked to bioactive compound Bias on non traditional biosynthetic pathways or novel resistance gene containing BGCs
Phylogeny mining	Reveals evolutionary patterns in biosynthetic pathways Minimizes rediscovery through targeted BGC mining Enhances understanding of BGCs' functional roles	Expensive and requires expert analysis Depends on the quality and completeness of reference genomic databases Predicted pathways or genes don't always yield natural products Bias on non traditional biosynthetic pathways
Large scale genome mining	Broadens discovery across diverse microorganisms and kingdoms Minimizes rediscovery through targeted BGC mining Provide insights into evolutionary pattern and biosynthetic capabilities	Expensive and requires expert analysis Depends on the quality and completeness of reference genomic databases Predicted pathways or genes don't always yield natural products Bias on non traditional biosynthetic pathways or novel BGCs
Metabolic engineering	Sustainable improvement and production of NPs Enhances chemical diversity through engineered biosynthetic pathways Precise modifications for novel NPD	Expensive laboratory settings and requires expert analysis Limited host compatibility Pathway alterations may disrupt cellular metabolism
Analogues discovery strategies	Expand chemical diversity Modify to enhance the NP pharmacological properties Reduces time and resources	Modification as not always as expected Require extensive screening to optimize the modification In many cases sophisticated techniques is needed Often requires advance techniques and expertise
AI-powered strategies	Several tools or pipeline or protocols uncovered the novel compounds or BGCs Hands in prediction reduces the cost of experimentation Easy, automatic and accurate the discovery	Model accuracy relies on dataset quality and algorithms AI tools without web servers need specialized expertise AI predictions require experimental validation, often yielding unexpected results

NP: Natural Product; NPD: natural Product Discovery; BGC: Biosynthetic Gene Clusters; AI: Artificial Intelligence

## Advanced culturing techniques and untapped resources or poorly cultivated organisms exploration

It is well-established facts that only 0.1–1% of natural microorganisms can be cultivated in standard lab conditions, with roughly 75% of bacterial phyla lacking cultured representatives [165, 166]. Many microbes “the uncultivated microbial majority” remain uncultivable due to unknown nutrient needs, specific environments, and symbiotic dependencies. Some grow slowly, rely on other species, enter dormancy, or thrive in extreme habitats beyond standard lab conditions [167].

High-throughput culturing techniques (HTCT) have recently begun to improve the cultivation of slow-growing and metabolically talented yet uncultivated microbes, surpassing classical methods limitations (see Sect. 2 and Table 3). HTCT such as micro well and microfluidic device [168], GALT prospector [169] and QPix platform [170] streamline cultivation by miniaturing and automating isolation. When coupled with hyphenated techniques such as IMS [171] and dereplication pipelines [121], these platforms can track both microbial growth and NPs production. Although, the GALT Prospector and QPix excel at handling difficult-to-culture microbes, such as rare human gut bacteria, their use in NPs-focused metabolomics remains underdeveloped [169, 170]. In contrast, miniaturized micro bioreactor systems (e.g. MATRIX 24-well microreactor format) have become increasingly common in NP discovery efforts, enabling scalable cultivation. Using this miniature fermenters, the Capon group uncovered several rare and structurally novel scaffolds, including the 2,6-diketopiperazine derivatives noonazines A-C, the azaphilone noonaphilone A from *Aspergillus noonimiae* CMB-M033980 [172], as well as anthelmintic polyketides goondapyrones A–J from *Streptomyces* sp. S4S-00196A1081 [173]. In addition, high-throughput dilution-to-extinction cultivation and behaviour-based models that mimic specific habitats have been employed to isolate a broad range of rare and underrepresented microbial taxa[174, 175]. These strategies have significantly advanced our understanding of microbial ecological niches. A notable example is the discovery of proteorhodopsin and its presence in *Pelagibacter ubique*, which underscores the value of accessing and studying culturable microbial isolates[176, 177]. Figure 3D provides an overview of additional modern cultivation techniques alongside representative microbial examples. At the simplest level, miniaturized micro-well culture systems and diffusion chambers allow parallel testing of growth conditions. Microfluidic devices further shrink volumes and increase throughput, while in situ cultivation platforms such as Ichip, C-chip, iTip, and SlipChip, replicate natural ecological niches by permitting environmental nutrients and signaling molecules to diffuse into isolated micro chambers [178].

Single-cell isolation techniques, including fluorescence-activated cell sorting [179], Raman-activated cell ejection [180], harness fluorescence intensity of unique indicators like metabolic activity, resistance profiles, and Raman spectral signatures to target individual microbes from complex consortia, respectively. Notably, the iChip was instrumental in the discovery of teixobactin, a novel antibiotic produced by *Eleftheria terrae*, a soil bacterium that had previously eluded cultivation [181].

**Fig. 4:**
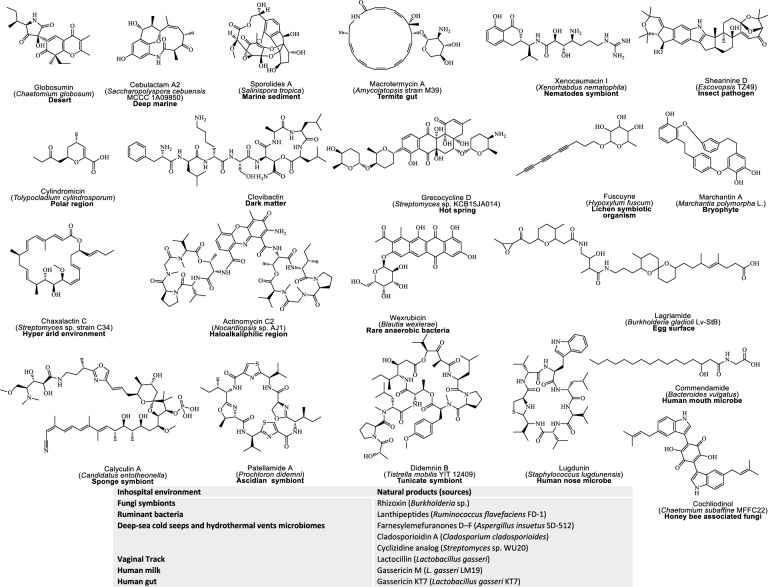
Representative examples of natural product from untapped and exotic environments. Each chemical structure is accompanied by its name, and source organism (provided in parentheses), along with the organism’s exotic habitat, which are highlighted in bold

Despite outstanding track records in innovative culturing techniques and NGS, approx. 80% of microbial sources remain underexplored [166]. To wrestle this difficulty, recent efforts have begun to chip away at this “microbial dark matter” by applying high-throughput culturing improvements such as droplet micro reactors, membrane-separated co-cultures, and hyphenated analytics, to activate silent biosynthetic gene clusters in neglected taxa and in-hospital niches [182]. These strategies have already yielded complex polyketides, nonribosomal peptides, polycyclic terpenes, rearranged steroids, and hybrid metabolites from once-inaccessible strains [183–185]. Additionally, Fig. 4 highlights several classical NPs that isolated from rare or extremophile microorganisms thriving in unusual or extreme environments, each paired with its unique environmental source and bioactive scaffold.

**Fig. 5:**
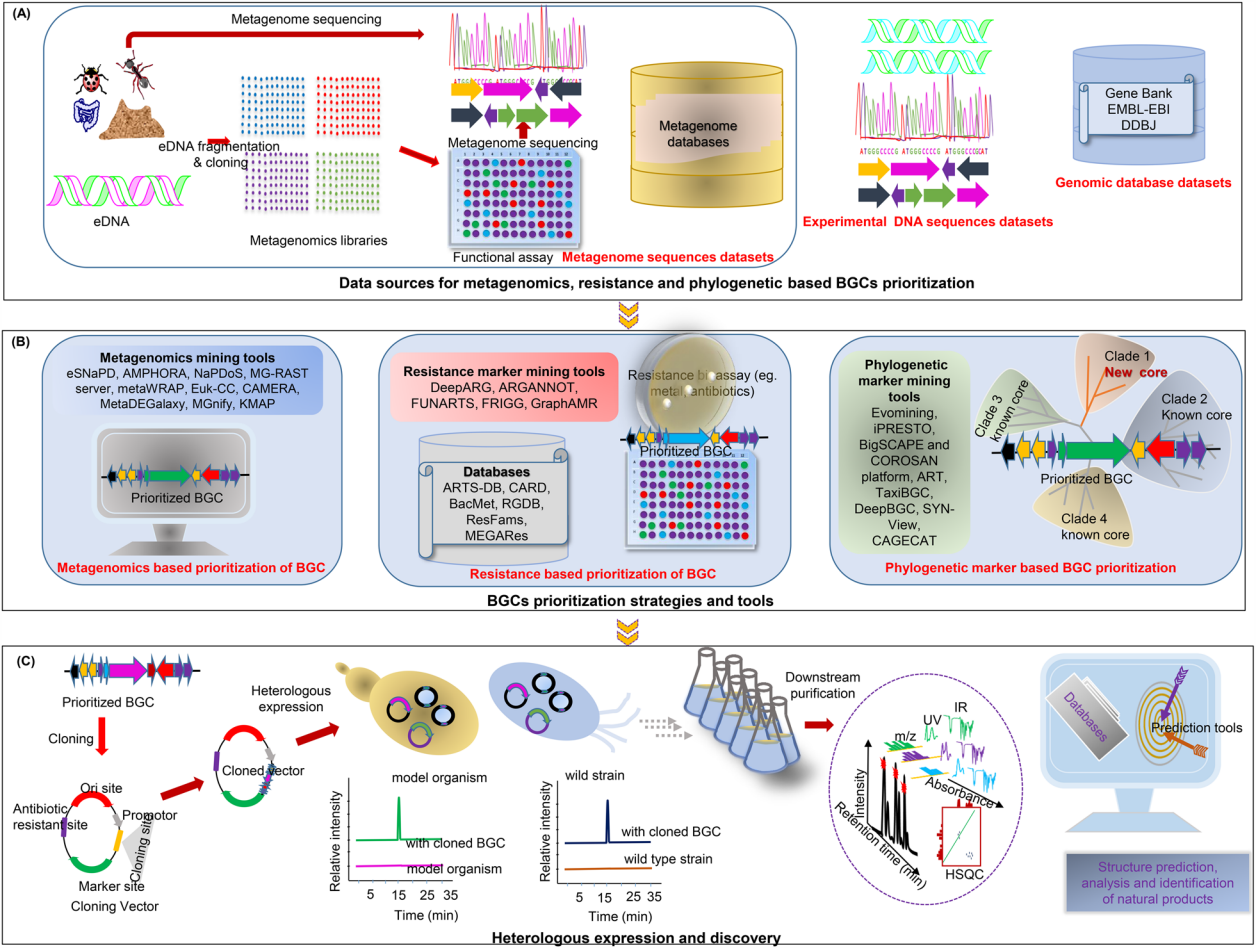
Schematic workflow and tools for genomic strategies in natural product discovery. Phylogenetic based mining, metagenomics based mining, and resistance gene based mining **A** Data sources; **B** Mining tools and databases and **C** Cloning, heterologous expression and identification of metabolites)

Beyond, culture-based methods, breakthroughs in culture-independent workflow (Fig. 5A**)** have enabled rare bioactive natural products characterization, while multiomic strategies have expanded access to untapped microbial resources, presenting an exciting frontier NPD [186] (Fig. 4). Despite these outstanding achievements in microbial culturing systems, many of these innovative techniques remain underutilized in NPD. The modernization of culturing methods has led to impressive strides in detecting and producing diverse NPs, yet, accessibility issues and implementation complexities hinder their full potential. Many natural product researcher find these emerging technologies out of reach, often due to limitation in resources, infrastructure, and technical expertise.

## Genomic and metagenomics innovations driving natural product discovery

NPs exhibit immense chemical diversity, yet their biosynthetic machinery is often highly conserved. Biosynthetic core enzymes typically share significant amino acid sequence similarity, enabling researchers to screen genomic data for specific biosynthetic genes that encode key enzymatic functions using genome mining tools [187]. Unlike bioactivity-guided isolation strategy, genome-based approaches are highly specific but do not provide immediate insight into a compound biological activity. In particular, in genome inspired discovery, researchers first identify candidate BGCs, benchmark enzyme domains against known pathways, and then experimentally probe the function and chemical output of these clusters [188]. Consequently, this strategy also led to the characterization of biosynthetic enzymes from uncultured microorganisms and cryptic BGCs that catalyze novel and exceptional chemistry, potentially linked to metabolites that are still poorly understood or entirely unknown [189].

Additionally, technological upgrade in sequencing have fueled this genomics driven NPD renaissance. Long read platforms (e.g. PacBio SMRT, Oxford Nanopore MinION), and high-throughput shorts reads (e.g. Illumina) have enabled rapid, high-quality, and cost-effective whole-genome and metagenomics assemblies [190]. At the same time, genomic studies have uncovered that bacteria, fungi, and even complex organisms possess a far greater biosynthesis capacity than lab experiments typically indicate [18], likely due to issues such as gene silencing and low metabolite yields. Unlocking these hidden compounds requires targeted- strategies or stimuli to activate silent or weakly expressed BGCs [191]. Nonetheless, key challenges such as identifying and prioritizing promising BGCs, effectively switching them on, and linking each BGC to its metabolite exists. To overcome these bottlenecks, a range of bioinformatics pipelines, genome-mining algorithms, integrative databases and online resources has emerged (Table 1). These resources enable researchers to identify BGCs, predict their chemical outputs, and rapidly dereplicate known compounds. Tools such as antiSMASH, and BiG-SCAPE facilitate the annotation, clustering, and prioritization of BGCs, while platforms like MIBiG and NPAtlas provide reference datasets for comparative analysis. When integrated with metabolomics workflows and interactive dashboards as detailed in Table 1, these platforms streamline NP discovery by reducing redundancy, boosting novelty detection, and directly correlating genomic predictions with LC–MS data [192].

Chronologically, evolution-based phenotypic characterization (1940–1970s), knowledge-based approaches (1970–1990s), computational-based approaches (1990s to early 2000s), and genome-AI integrated approaches (mid-2000s onward) indicate the trend and progress of genome mining [193]. Despite its remarkable progress, connecting genetic information to the enzymatic and structural characterization of the encoded NPs remains a significant bottleneck (roughly 90% of Actinobacterial BGCs are still uncharacterized) [18]. To bridge this gap, large-scale data-driven bioinformatics platforms now multiplexed with hyphenated analytical datasets (e.g. iSNAP) [194], microcrystal electron diffraction techniques (MicroED) [195], and machine- and deep learning architectures [39]. This enables the characterization of novel molecules, chemical building blocks, biosynthetic signatures, tailoring enzymes, and biosynthetic pathways [43], by targeting chemical features, families, entire BGCs or only domains while minimizing labor-intensive efforts and rediscovery, and maximizing NP diversification [196]. For example, *Kim *et al*.* (2021) employed genome mining to pinpoint the *icc* BGC in *Penicillium variabile*, leading to the discovery of a novel 2-pyridone natural product, Py-469 and solved the structure via MicroED, demonstrating the synergy between genomic prediction and structural elucidation [195]. Likewise, Li et al. [195] identified a previously cryptic csp cluster in the anaerobe *Clostridium roseum*; through gene knockouts and heterologous expression, they confirmed its role in biosynthesizing the new clostyrylpyrones. Moreover, Liu et al. (2025) developed NegMDF, a workflow integrating mass defect filtering and bioinformatics to link BGCs with metabolite ions under negative ionization. Applied to *Streptomyces cattleya* NRRL 8057, it rapidly identified 22 polyketides, including rare tetronate-containing cattleyatetronates. Collectively, these case studies illustrate how coupling advanced bioinformatics, structural techniques, classical genetics, and metabologenomic can unlock the vast chemical potential encoded in silent BGCs.

Metagenomics, first coined in the late 1990s, enables culture-independent exploration of microbial genomes, including those from individual cells, across a wide range of extreme and untapped environments such as Polar Regions, deep-sea ecosystems, and the gut microbiome. Over the past two decades, its robustness has powered discovery of novel BGCs and pathways in uncultivable microbiomes, often by directly cloning environmental DNA libraries into heterologous hosts for expression [186, 197, 198]. Standard metagenomics protocols (Fig. 5A employ functional screening of eDNA or shotgun sequencing followed by BGC prediction. Indeed, a crucial pillar in NPD, this approach faces challenges such as labor-intensive procedures for constructing and screening large libraries, dealing with non-genomic DNA during metagenomics bin assembly, and masking low-abundance microorganism DNA, making it difficult to accurately link metabolites or genes to phylogenetic knowledge [199, 200].

To combat these issues, targeted phylogenetic metagenomics that focuses on capturing large BGC fragments by PCR amplification of conserved biosynthetic domains (e.g. AT, KT, ACP) directly from the eDNA [201]. And, parallel strategies screen for entirely novel biocatalysts in metagenomes using activity-based assays [202] and leverage computational tools for in silico prioritization. More recently, modern consortiums and web-based annotation platforms such as MetaSU for urban environments [203] and MetaHIT for the human gut [204] now map AMR markers and discover new BGCs global scale.

Additionally, single-cell metagenomics adds another dimensions by sorting individual cells from environmental samples via FACS [205] or microfluidic droplet platforms [206] before whole-genome amplification and sequencing, unlike traditional metagenomics, which sequences pooled DNA from entire communities. This approach enables precise bioinformatics assignment of genes to specific microbes, making it especially useful for studying uncultivable organisms. By linking metabolites and genetic pathways to phylogenetic data, it offers deeper insights into microbial diversity and biosynthetic potential serving as a powerful complement to community-level sequencing methods [207].

**Fig. 6:**
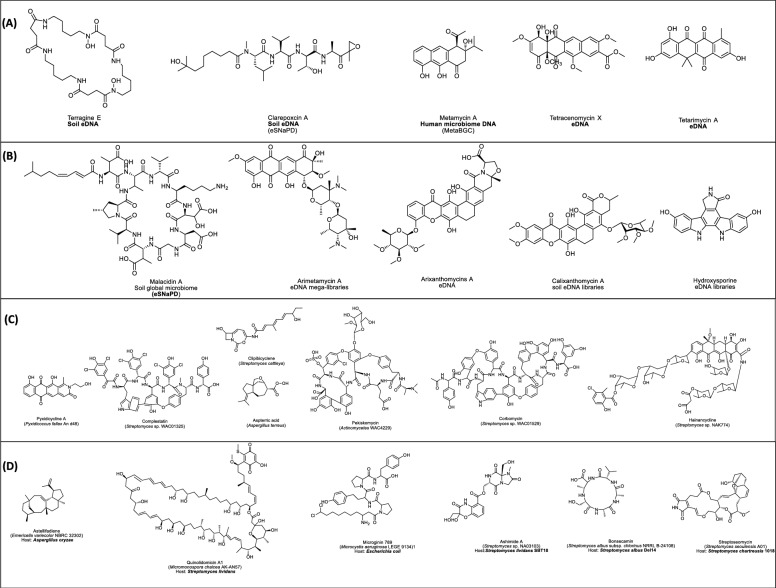
Examples of representative examples of natural products (NPs) discovered with different genomic mining approaches. **A** Examples of NPs discovered via metagenome mining **B** Examples of NPs isoalted by resistance gene mining, **C** Examples of NPs discovered via phylogeny-inspired mining and **D** heterologous expression based discovered NPs. Each chemical structure is provide with its name, sources or tools/expression system applied

Traditional and modern single-cell metagenomics techniques are powerful tools for exploring uncultured microorganisms in environmental samples or eDNA. These approaches have enabled the discovery of diverse NPs like apratoxin A (e.g. konbamides, nazumamide A, keramamides, cyclotheonamide), misakinolide A and theonellamides [200]. Additionally, Fig. 6A–B highlights additional examples, discovered through metagenomics approaches, particularly from uncultured or symbiotic microorganisms. These discoveries underscore the power of both traditional and single-cell metagenomics in accessing cryptic BGCs and expanding the chemical diversity.

## Large-scale genome mining in modern genomics: exploring resistance-gene or phylogenetic relationship

### Resistance gene based genome mining

Prioritizing BGCs for targeted production of NPs with desired bioactivities remains a complex challenge in genome mining. Beyond core synthesis enzymes, BGCs often include genes for transport, modification, and self-resistance. Self-resistance clusters have evolved to neutralize environmental antibiotics via detoxification, efflux pumps, target binding or modification, and even horizontal gene transfer [208]. Exploiting these resistance genes as “molecular beacons” provides a powerful genome-mining strategy: by flagging clusters that harbor self-resistance determinants, researchers can predict NP modes of action and narrow down BGCs for further study [64, 209]. For example, Panter et al. [210] screened myxobacterial genomes for pentapeptide-repeat proteins homologous to the cystobactamid resistance mechanism and discovered the pyxidicyclines, a new class of type II polyketides with a nitrogen-containing tetracene core. These compounds inhibit bacterial topoisomerase IV (IC₅₀ 1.6–6.25 μg/mL) and human topoisomerase I, and exhibit potent cytotoxicity against HCT-116 cells (MIC 0.06 μg/mL).

Additionally, modern bioinformatics platforms such as ARTS-DB [64], CARD [65], DeepARG [66] automate the identification, annotation, and functional analysis of resistance genes from genomic and metagenomics data, accelerating resistance-guided NP discovery. As listed in Table 1F, multiple databases and tools support this workflow; and Fig. 6B highlights notable NPs uncovered through resistance-gene directed mining.

### Phylogenetic relationship based genome mining

Complex evolutionary and metabolic processes such as insertions, deletions, duplications, rearrangements, and both vertical and lateral gene transfer shape multigene BGCs, which encode proteins for core molecule synthesis, diversification, regulation, and transport [211]. Conserved enzyme domains such as ketosynthase alpha (KSα) and beta (KSβ) often evolve through concerted mechanisms, originating from within BGCs, from other clusters, or even from central metabolism [212, 213]. By building phylogenies around these domains, researchers can pinpoint divergent or novel sequences and then mine representative genomes for the underlying chemistry. For example, Mullins et al. (2021) reconstructed a phylogeny of alkyne and polyyne biosynthetic cassettes, allowing them to identify distinct phylogenetic clades of interest. By mining representative genomes from these clades, they discovered previously uncharacterized polyyne BGCs. Notably, within the Gammaproteobacteria clade; led to the discovery of a novel polyyne “protegencin” produced by *Pseudomonas protegens* (formerly *P. fluorescens*) strains Pf-5 and CHA0. Similarly, *Deng *et al*.* (2025) discovered mandimycin, a polyene antifungal antibiotic, using a phylogeny-guided platform based on conserved mycosamine-transferring glycosyltransferases, a modification enzyme from *S. netropsis* DSM 40259. Mandimycin showed potent, broad-spectrum activity against multidrug-resistant fungal pathogens, with MICs ranging from 0.125 to 2 μg/mL [214]. Additionally, Table 1 summarizes automated bioinformatics tools that automate phylogeny-based NPD and outlines their specific functions and features, and Fig. 6C presents additional landmark phylogeny-guided discoveries.

Recently, beyond single-cluster screens, large-scale pan-genomic mining of closely related entire taxa [215], genus [216], special niche microbiome (e.g. Ocean, acid mine drainage, soil) [217, 218] and specialized strain collections [219] has revealed thousands of BGCs and unraveled their evolution relationships. To date, diverse microbial sources including Shark Bay microbial mats, *Virgibacillus*, *Cyanobacteria*, the swine gut microbiome, entomopathogenic nematode-symbiotic bacteria, *Streptomyces*, *Saccharomonospora*, *Burkholderia*, marine prokaryotes, turtle ant gut-symbionts, *Penicillium*, and archaea have been mined for NPs. These mining efforts systematically investigate thousands of BGCs, revealing complex evolutionary relationships and mechanisms [220]. In particular, setting biosynthetic genes or specific functional domains within the BGCs into a phylogenetic relationship with known sequences to track the proximity and outliers to the known sequences [221] predicts and prioritizes substructures and full chemical structures from BGCs, often facilitated by recent automatic platforms.

Notably, automatic platforms, EvoMining for enzyme family evolution [222], DeepBGC for machine learning based BGC detection [223], TaxiBGC for taxon-guided mining [215] and BiG-SCAPE/CORASON for chemical-family clustering [36, 224] predicts and prioritizes substructures of both substructures and full chemical scaffolds. NaPDoS, for instance, a bioinformatics tool to study the phylogenetic relationship studies of PKS and NRPS domain. Researchers used the tool to analyze ketosynthase (KS) domains from the marine actinomycete genus *Salinispora* [225], and Cruz-Morales et al. [226] applied EvoMining to actinobacterial genomes and reconstructed the evolutionary history of 23 enzyme families, uncovering a previously unrecognized BGC in *Streptomyces coelicolor* and *S. lividans* responsible for producing arseno-organic metabolites.

Focusing on candidate BGCs associated with resistance genes offers a strategic advantage, as the resulting compounds are more likely to possess biological activity. This approach is grounded in the idea that the compound’s mechanism of action mirrors the native function of the resistance determinant, thereby enabling for more accurate predictions of bioactivity. In contrast, phylogeny-based studies expand search insights by linking to habitat-specific, taxonomic (species/genus), behavioral, or evolutionary traits to novel chemistries, beyond relying solely bioactivity-driven selection alone. Together, resistance- and phylogeny-guided mining form complementary pathways that accelerate the discovery of NPs with both new structures and targeted bioactivities.

## Downstream applications of genome mining for natural product discovery: cloning, heterologous expression and CRISPR-Cas

### Cloning and heterologous expression

**Fig. 7:**
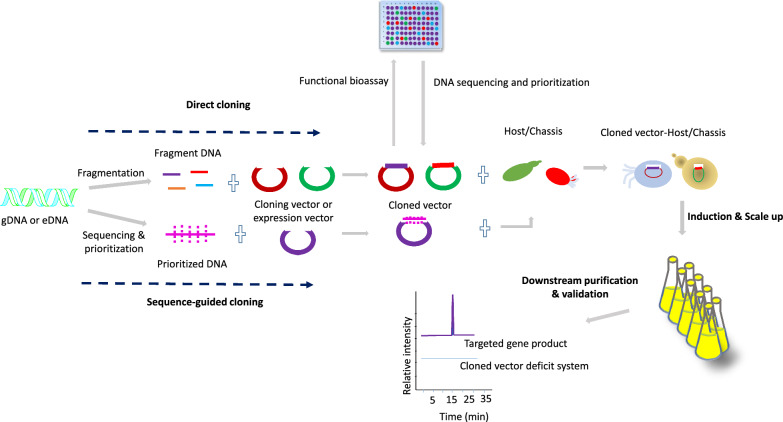
Schematic workflow for cloning and heterologous expression in natural product discovery

Beyond conventional methods like phenotype screening, co-culture, and elicitor-based assays, an emerging strategy involves cloning and heterologous expression of BGCs within optimized host organisms. Cloning and heterologous expression systems are indispensable and closely linked for overproduction and NPs discovery. First introduced in the 1960s, scaled up with recombinant DNA advancements in the 1990s, enabling efficient biosynthetic gene expression in model organisms to optimize titer, rate, and yield ((TRY), including NCEs discovery [76, 227]. In particular, this technique involves cloning and expressing engineered gene clusters often sourced from untapped and extreme environments [76, 228]. In addition, it enables functional expression of previously silent or uncharacterized pathways that may encode valuable bioactive compounds or NCEs [76, 226]. Figure 7 illustrates the foundational workflow involved in cloning for NPD: prioritize and prepare DNA fragments, choose suitable cloning vectors and assembly methods, then select and engineer host strains to functionally express silent or novel pathways.

Early cloning vector systems such as cosmids and fosmids accommodated inserts up to ~ 50 kb, but many BGCs fall in the 100–350 kb range. To handle these larger clusters, researchers developed high-capacity vectors P1-derived artificial chromosomes, bacterial artificial chromosomes, and yeast artificial chromosomes, capable of stable maintenance of large DNA inserts for complex biosynthetic pathways and expression. Detailed information on these vectors is available in specialized review article [229]. While *E.coli* followed by *Streptomyces*, *Schizosaccharomyces, Saccharomyces* and *Aspergillus* were the contemporary heterologous expression system. Nevertheless, their applications remains limited by incompatibilities in transcriptional regulation, codon usage, and lack of post-translational modifications. Moreover, other challenges include insufficient precursor/co-factor availability, product toxicity, and poor biosynthetic assembly of NCEs. Eukaryotic gene clusters present additional hurdles, requiring intron splicing and strategic insertion of promoters and terminators for functional expression [230]. Of late, to overcome these limitations, diverse engineered streptomycetes such as *S. avermitilis, S. venezuelea* and emerging non-*Streptomyces* hosts, for instance, *Myxococcus xanthus*, *Yarrowia lipolytica*, *Burkholderia thailandensis* strain E264, *Bacillus subtilis*, *P. putida* have been the choice of chassis for the HES for more compatible expression environments [231, 232].

Similarly, selecting an appropriate recombination method is equally critical in success of cloning-based approaches for NPD. The choice of DNA assembly method influences the efficiency, accuracy, and scalability of DNA assembly particularly when dealing with complex BGCs. Classical in vitro DNA assembly methods, such as restriction enzyme-mediated digestion and ligation, rely on specific recognition sequences, limiting design flexibility and making them inefficient for assembling large or multiple DNA fragments. These approaches often leave behind unwanted scar sequences and require labor-intensive steps like digestion, purification, and transformation making them ill-suited for high-throughput or modular synthetic biology applications [229]. In contrast, modern recombination-based strategies including Gibson Assembly, DiPaC, CATCH, and CCTL enable seamless, scarless assembly without dependence on restriction sites, while enzyme-independent approaches offer additional design versatility. Additionally, in vivo approaches such as phage recombinase-mediated homologous recombination in *E. coli*, TAR cloning in yeast, and site-specific integrase systems (e.g. Cre/loxP, ΦC31, ΦBT1) allow efficient and precise capture of large DNA segments directly within host cells, further expanding the toolkit for complex NPD [233, 234].

#### Direct cloning

Direct cloning (Fig. 7), where fragmented environmental or genomic DNA is immediately inserted into expression vectors without prior sequencing. These vectors are then used to construct large libraries, which are screened through in vivo or in vitro functional assays to identify bioactive compounds or novel biosynthetic pathways. This strategy is particularly valuable when genomic information from the native host is poorly characterized or unavailable. By bypassing the need of prior sequence information, sequence-independent approaches enable comprehensive exploration of a sample genetic content, often increasing the likelihood of capturing entire BGCs and uncovering structurally novel NPS.

However, direct cloning faces notable constraints. Random insertion of DNA fragments may yield incomplete or nonfunctional BGCs, and large clusters frequently exceed standard vector capacities. In addition, native regulatory elements may be incompatible with the heterologous host, leading to inefficient transcription and translation. Moreover, functional screening is labor-intensive and prone to false negatives due to the low frequency of bioactive clones.

To overcome these limitations, careful pairing of cloning systems with high-throughput screening assays is essential. In place of traditional *E. coli*, researchers increasingly opt for phylogenetically closer expression hosts such as *S. lividans*, *S. albus*, and *P. putida.* Additionally, introducing regulatory elements such as sigma factors into heterologous hosts can significantly enhance the expression of target biosynthetic genes. Figure 6 highlights several classical examples of NPs discovered through both direct cloning and the use of advanced heterologous expression systems.

#### Sequence-guided cloning

Sequencing-guided cloning begins with the sequencing of environmental or genomic DNA, followed by bioinformatics analysis to identify and prioritize BGCs with high potential for producing bioactive compounds [235]. Tools such as antiSMASH 7, PRISM 3, ARTS, and BiG-SCAPE assist in predicting BGCs functions and selecting promising candidates [236] (see Table 1B–C). Once prioritized, the targeted gene clusters are cloned into suitable expression vectors, enabling direct functional characterization and NPD. This approach bypasses traditional random library construction and broad screening by focusing efforts on specific, high-value clusters, streamlining the discovery of novel NPs.

A notable example is the discovery of cadasides A and B, acidic lipopeptides, acidic lipopeptides identified by Wu et al. [237], sequenced nonribosomal peptide synthetase (NRPS) adenylation domains to pinpoint and clone the *cde* BGC from calcium carbonate rich soil samples. Their analysis revealed a correlation between cadaside-like domain abundance and specific geochemical environments, enabling targeted recovery and successful expression of the cluster. Similarly, Zhao’s group cloned and heterologously expressed 105 BGCs in *Streptomyces lividans* TK24. Their use of the Antibiotic-Resistant Target Seeker tool to prioritize clusters based on self-resistance gene markers led to eight novel bioactive compounds from five productive BGCs, with efficient cloning achieved using the CAPTURE platform [238].

Together, Cloning and HES have revolutionized NPD, enabling the scalable production of novel compounds and unlocking hidden chemical treasures (For instance, Fig. 6D). Yet, research on selecting the optimal cloning vector, heterologous host for maximum natural product yields remains a significant challenge due to the differences in metabolic scaffolding among hosts, such as sporulation, machinery tools, biofilm formation, and autolysis.

### CRISPR-Cas9 system

The CRISPR-Cas9 system, originally derived from bacterial adaptive immunity and conceptually identified in the late 1980s, became a programmable genome-editing tool in the early 2010s. Its emergence revolutionized genetic engineering and has since played a crucial role in NPD, especially for targeted genetic disruption. By enabling precise modifications such as gene knockouts, knock-ins, and knockdowns, CRISPR-Cas9 allows researchers to screen specific phenotypes and manipulate biosynthetic and metabolic pathways to enhance NPs yields and unlock new compounds [239, 240].

**Fig. 8:**
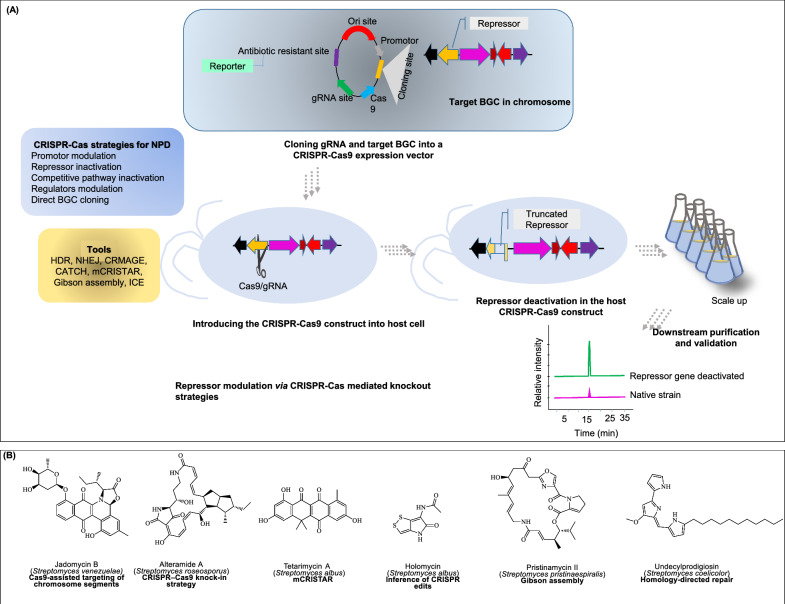
CRiSPR-Cas for natural product discovery. **A** Workflow of knockout strategies of CRiSPR-cas strategies. **B** Examples of discovered metabolites via CRiSPR-cas strategies

Earlier genome editing technologies such as meganuclease I-SceI [241], zinc-finger nucleases [242], and transcription activator-like effector nucleases [243] formed the basis for CRISPR-Cas9. This technology marked a significant milestone in genomic biology [244], enabling applications such as targeted gene regulation, epigenetic perturbation, chromatin manipulation, and live cell chromatin imaging paradigm [245]. Figure 8A illustrates the workflow of the CRISPR-Cas knockout strategy used in NPD.

Importantly, CRISPR-Cas systems are not primarily used to discover entirely new BGCs, but to investigate, optimize strain improvement and scalable production function of known ones. Through precise genome editing, CRISPR-Cas enables researchers to dissect the roles of individual genes within biosynthetic pathways, identifying key enzymes, regulatory elements, and metabolic bottlenecks. More importantly, it allows for the fine-tuning of these pathways by knocking out inhibitory genes, activating silent clusters, or engineering promoter regions, ultimately boosting the yield and efficiency of NPs biosynthesis [246, 247]. Consequently, CRISPR-Cas9-based genome editing is extensively used for genetic and metabolic manipulation of bacteria, yeast and plants to discover hidden chemical treasure troves [248].

Several hallmark studies illustrate the breadth of CRISPR-Cas applications in NPD. For example, *Bushin *et al*.* [249] identified a quorum-sensing, regulated RiPP gene cluster from *Streptococcus* cloned using CRISPR-assisted and expressed the cluster in *S. albus*, leading to the production of of streptosactin, a sactipeptide with unique crosslinks and strong antifungal activity against *Candida albicans* and *Aspergillus fumigatus*. *Ameruoso *et al. [250] developed CRISPR interference (CRISPRi) and activation (CRISPRa) systems in *Streptomyces venezuelae* to regulate transcription of silent biosynthetic gene clusters. By modulating key regulatory networks, they successfully activated the jadomycin B cluster, leading to natural product synthesis previously undetectable under standard conditions. Likewise Peng et al. [251] constructed a CRISPR-dCas9 system in *Myxococcus xanthus*, enhancing epothilone B production. By fusing dCas9 with a transcriptional activator and targeting promoter regions of the 56-kb biosynthetic gene cluster, they achieved a 6.8-fold yield increase. These three examples illustrate the successful application of CRISPR-Cas technology in distinct areas of natural product research: the discovery of new compounds, activation of silent gene cluster and the scalable production of valuable metabolites. Figure 8B presents examples of NP modulated using CRISPR-Cas technology.

Despite its versatility, CRISPR-Cas9 faces limitations such as off-target effects and strict PAM sequence requirements for SpCas9 from *Streptococcus pyogenes*, which is widely used in bacteria and archaea [248]. To overcome these challenges, improved Cas proteins such as saCas9 and fnCpf1, have been developed or engineered, offering greater specificity and broader target accessibility [252]. For instance, in a recent study by Zhou et al. [253], researchers addressed the limitations of class 2 CRISPR systems like Cas9, which often underperform in *Streptomyces*, by repurposing the native type I-E CRISPR system into transcriptional regulators (CRISPRi and CRISPRa). Applied across nine diverse *Streptomyces* strains, these tools successfully activated 13 of 21 cryptic biosynthetic gene clusters, revealing five new NPs: one polyketide, one RiPP, and three alkaloids.

## Advances in genomic mining-based metabolic engineering for natural product discovery

Historically, metabolic engineering aimed to enhance the TRY of target molecules using cost-effective nutrients, particularly in pharmaceuticals production [254]. The biosynthesis of NPs, however, is influenced by a variety of factors, including growth conditions, carbon source availability, and complex layer of genetic regulation. These factors pose significant challenges when attempting to clone and fine-tune large BGCs, particularly because many of these clusters remain genetically intractable, exhibit poor expression under laboratory conditions, and are often silent, meaning they are not naturally expressed due to unknown regulatory mechanisms, diverse secretion systems, and intricate metabolite profiles. Modern bioinformatics tools and omics knowledge, including “metabolic engineering” and “BGC refactoring” have addressed some of these limitations of NPD exploration and overproduction [230, 255].

Over the past decade, its application has expanded to manipulate biological systems to build alternative pre-programmed systems harboring features for targeted compounds biosynthesis. A key focus has been on the prediction and reconstruction of metabolic pathways using available genomic and metabolic data. Recently, three prominent tools that have emerged to support this effort include GENREs, BiGMeC pipeline, and Galaxy-SynBioCAD portal. GENREs provides a quantitative framework that integrates genomic, biochemical, and phenotypic data to model and analyze biochemical reactions systematically across defined metabolic categories [256]. While, the BiGMeC pipeline, on the other hand, automates the reconstruction of metabolic pathways specifically associated with PKS and NRPS gene clusters, streamlining NPD from complex genomic datasets [257]. Galaxy-SynBioCAD portal facilitates the creation of strain libraries optimized for producing specific chemical compounds, encompassing the entire workflow from selecting target molecules and host strains to designing and engineering complete metabolic pathways [258] (Table 1D–E). Additionally, recent efforts have focused on constructing metabolic pathways by leveraging time-series multi-omics data to capture metabolic dynamics [259], alongside analyzing flux distributions [260] is discussed elsewhere.

Basically, metabolic engineering in NPD aims at, (i) enriching the target compounds TRY [261], (ii) modifying NPs scaffolds for improved bioactivity [262], (iii) engineering and expressing the BGCs from diverse sources (e.g. marine microorganism [263], endosymbionts [264]), and (iv) dissecting unsolved biosynthesis pathways [262]. Although, the term “*metabolic engineering”* is relatively generic, its core principles have been consistently recognized across the literature and are summarized in Table 4.

**Table 4:** Major metabolic engineering strategy

Metabolic engineering techniques	Description		Natural product example	Key strategy	References
Host engineering	Either the engineering of a wild-type or native organism, or a strain already engineered for specific production purposes	Kanglemycin A		The refactored KglA biosynthetic gene cluster was introduced into *Streptomyces coelicolor*, followed by deletion of native BGCs to minimize metabolic competition. Introduced *rpoB* and *rpsL* mutations to enhance transcription and translation, while central metabolism was reprogrammed to increase precursor flow toward polyketide synthesis	[265]
Chassis engineering	Re-engineering of a previously optimized host to serve as a versatile chassis for broad biosynthetic applications	Chloramphenicol (40X increase yield)		Re-engineering of *S. coelicolor* by deleting four native BGCs, introducing *rpoB*/*rpsL* mutations to boost gene expression, and integrating the chloramphenicol BGC from *Streptomyces venezuelae*	[266]
Bioprocess engineering	Designing and optimizing bioreactors, fermentation conditions, and downstream processing	Camptothecin (1.5X increase yield)		Plackett–Burman design was used to optimize medium components such as glucose, dextrin, salicylic acid, serine, and cysteine, glutamate, and resin adsorbents	[267]
Reaction engineering	Designing, and optimizing chemical reactions within reactors	Artemisinin		In engineered *Saccharomyces cerevisiae*, bioreactor parameters including pH, temperature, and oxygen levels were tightly regulated to enhance enzyme activity and maximize artemisinic acid production; this precursor was then chemically converted to artemisinin via a photo oxidation process requiring light, oxygen, and controlled thermal conditions	[268]
Pathway engineering	Modification and optimization of metabolic or signaling pathways within a biological system	Jadomycin B		Used CRISPR interference (CRISPRi) and CRISPR activation (CRISPRa) to precisely modulate BGC gene expression within *S. venezuelae*	[250]
Enzyme engineering	Modification of enzymes to improve or alter their properties for specific enzyme	Novel variants of lipopeptides belonging to the iturin family		Engineering and modifying the substrate-binding pocket of acyl ligase domain of NRPS enzymes in *Bacillus* species, they altered the fatty acid chain length of iturin-family lipopeptides	[269]
BGCs refactoring	Redesigning and reconstructing NP biosynthetic pathways	Daptomycin (20.4 X increase yield)		Using CRISETR, a fusion of CRISPR/Cas9 and RecET recombination, researchers swapped native promoters for synthetic alternatives in the daptomycin BGC *S. coelicolor* to enhance production	[270]

Undoubtedly, the various metabolic engineering strategies outlined in Table 4 work synergistically to support either NP enhancement or NPD. Among them, BGC refactoring enables activation of silent pathways and diversification of NPs. It involves core biosynthetic genes modulation such as modular domain- deletion [271], addition or sequence alteration [272], shuffling [273]. For instance, in a recent study, the Calcott research group introduced over 1,000 unique metagenomics domains into a pyoverdine NRPS system, resulting in the production of 16 distinct pyoverdines as major products [274]. In contrast to single-module modifications, Song et al. [275] developed RedEx, a technique that enables the precise insertion or deletion of large DNA fragments within extensive polyketide synthase (PKS) clusters. Using this approach, they successfully replaced the C-21 ethyl group of the macrolide insecticide spinosad with a butenyl group, creating a new analog with modified structure. A complementary strategy focuses on engineering non-core biosynthetic elements such as promoters; regulatory genes (see Sect. 7.1–7.3).

**Fig. 9:**
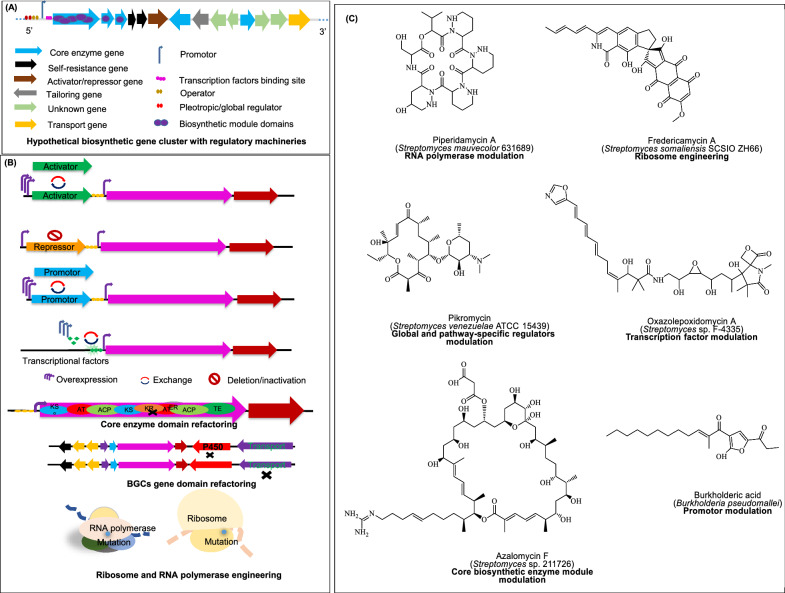
Metabolic engineering strategies for fine-tuning biosynthetic gene cluster domains **A** A schematic diagram for hypothetical BGC. **B** Different metabolic strategies applied to activate BGCs. **C** Examples of NPs discovered viz-metabolic modulation strategies. Each metabolite is provided with its name, source and strategy applied

Despite numerous innovative protocols advancing NP discovery and production, the formation of toxic intermediates remains a persistent challenge. These compounds can disrupt biosynthetic pathways and trigger feedback inhibition, posing significant barriers to pathway stability and productivity. The mechanisms underlying their generation and impact are still not fully elucidated. To address this issue in metabolic engineering, several strategies have been developed. These include enhancing host organism tolerance to toxic metabolites, integrating continuous flow bioreactors to facilitate real-time toxic metabolites removal, and fine-tuning regulatory elements such as promoters, ribosome binding sites, and terminators (Fig. 9A–C), to ensure balanced pathway expression and minimize accumulation of harmful intermediates.

### Post- and transcriptional regulatory features modulation

Modern bioinformatics analysis uncovers that over half of BGCs harbor regulatory genes, but the networks controlling BGC expression remain complex and not fully understood [276]. These regulatory proteins bind to DNA elements influencing transcription levels [277] and can be classified as global (e.g. N-utilizing factor G, A-factor-dependent protein A, CodY, LaeA, MilR_3_), epigenetic (Mehat, NnaB, rpdA) and pathway-specific (e.g. samR0484, gbnA/gbnB, tnmR1/tnmR3/tnmR7, ACE1, chal, SlnR, MonH/MonRI/MonRII) [278, 279]. For a deeper understanding of individual regulators roles in NPD, readers are referred to literatures on epigenetic-[280], fungal-[278] regulatory system. Over the past decade, engineering these regulators has markedly improved silent BGC activation and NP yields in *Bacillus* [281], *Streptomyces* [282], *Penicillium* [283] and *Aspergillus* [284]. Briefly, strategies for gene expression modulation include disrupting negative regulator genes [285], altering mRNA processing [286], inserting synthetic regulatory systems [287], and modifying epigenetic processes [280]. Although RNA interference based approaches for BGC expression modulation show promise, their application in NPD remains in its infancy and has so far enabled the discovery of only a few NPs (e.g. platensimycin and platencin [288], phomallenic acids A-C [288]).

Advanced high-throughput biosensor enabled screening systems have revolutionized the way we modulate regulatory networks in microbial hosts by directly coupling intracellular metabolite levels to easily measured signals. For example, transcription factor-based biosensors such as VanR-VanO vanillate sensor [289] and FRET-based and NADH/NAD + based ratio metric biosensor [290] allow dynamic monitoring and control of pathway intermediates and redox states in real time. When combined with automated liquid-handling platforms and microfluidic cultivation, these biosensors enable systematic optimization of host chassis, genetic constructs, and culture conditions, while also supporting high-throughput screening of vast regulator libraries to pinpoint variants that enhance target molecule production [291, 292]. Complementing these experimental advances, the Ligify software mines enzyme-reaction databases to predict transcription factors likely responsive to user-defined chemicals, thereby accelerating the design of bespoke biosensors for novel NPs [293].

### Promoter modulation

Promoters, small DNA segments located upstream of the 5′-ends of structural genes, enable RNA polymerase recognition and binding, thus controlling gene expression. These elements can be identified in silico using tools such as iProEP [294] and PromGER [295], and validated in vivo through techniques like ChIP-on-chip [296, 297] or electrophoretic mobility shift assays [298]. Because native promoters are often quiescent under laboratory conditions, they are prime candidates for modification by random- or site-directed mutagenesis, hybridization, error-prone PCR, and sequence randomization. Engineering or substituting these dormant promoters with well-characterized constitutive, host-specific, or inducible promoters has proven highly effective for activating cryptic BGCs and enhancing compound titers [299–301], including in actinomycetes, *E. coli*, cyanobacteria, and fungi [302]. For instance, Lin et al. [303] introduced the inducible promoters alcA and aldA, which are activated by alcohols, aldehydes, and ketones, to drive expression of the sartorypyrone BGC (*spy*) from *A. fumigatus* Af293 in the heterologous host *A. nidulans*, yielding twelve sartorypyrones (five known and seven novel).

Orthogonal synthetic circuits such as the Q-system from *Neurospora crassa* [304] or synthetic Tet-On/Off in *Aspergillus*[305] repurpose non‐native regulators to drive BGC transcription without perturbing host networks. For instance, harnessing the modular Q-system from *Neurospora crassa*, Lalwani et al. [304] engineered optogenetic circuits in *Saccharomyces cerevisiae* to achieve precise light-responsive gene regulation. These include two complementary platforms: OptoQ-INVRT circuits, which activate transcription in darkness, and OptoQ-AMP circuits, which trigger robust expression under blue light, yielding up to a 39-fold increase in gene activity for geraniol and linalool terpenoids production.

While diverse promoter libraries accelerate pathway regulation, they struggle to coordinate multi-gene expression in complex networks. To address this, researchers now pair promoter tuning with metabolic flux analysis [306], real time intermediate biosensor [307], mathematical model simulation [308], and AI-driven construct design [309]. For instance, in a recent study, Liu's team enhanced GlcNAc production in *B. subtilis* by integrating promoter tuning with a real-time GlcN6P biosensor and ADC-based feedback circuits. This dynamic regulation increased GlcNAc titers in a 15 L fed-batch bioreactor from 59.9 to 97.1 g/L (with acetoin) and from 81.7 to 131.6 g/L (without acetoin), demonstrating the robustness and scalability of promotor integrated approaches [310].

Recent advances in AI-based promoter modeling have harnessed deep learning and generative frameworks to predict and design synthetic promoters with precise strength, specificity, and regulatory features for diverse microbial hosts [309]. At the same time, CRISPR-Cas9 mediated promoter replacement enables exact, in situ swapping of native regulatory sequences with engineered or inducible promoters directly within biosynthetic gene clusters, dramatically improving transcriptional tuning [311]. In addition, tools like Easy Promoter Activated Compound Identification [312] uses in situ promoter exchange to selectively activate BGCs encoding NRPS, PKS, NRPS-PKS hybrids, or other BGC classes, yielding targeted NPs. Applied to *Xenorhabdus* mutants, this approach uncovered antiprotozoal metabolites including fabclavines, xenocoumacins, xenorhabdins, and PAX peptides [313]. Together, these integrated approaches deliver efficient, dynamic transcriptional control and underscore the transformative power of promoter engineering in NP biosynthesis.

### Editing ribosome binding sites (RBSs) and terminators

Transcription regulation of BGCs is complex and influenced by various internal and external factors [314]. At the transcriptional level, synthetic transcription factor decoys can be tuned via copy number or decoy‐site sequence to control native and heterologous gene expression, driving a 16-fold increase in arginine production in *E. coli* [315]. At the translational level, the ribosome-binding site’s translation initiation rate (TIR) is critical for balancing multi‐gene operons; engineering RBS nucleotides through design and HTS of synthetic [316] or pre-characterized libraries [317] fine-tunes TIR to optimize biosynthetic output. For example, targeted mutations in the RBSs of *vioB*, *vioC*, *vioD*, and *vioE* relieved bottlenecks in violacein biosynthesis, yielding a 2.41-fold titer increase in *E. coli* [315]. Similarly, constructing a 5′-UTR library by random base insertions on truncated upstream sequences boosted riboflavin titers 2.09-fold in *B. subtilis* [318].

Further improvements in transcript stability and pathway efficiency come from terminator engineering [319], sigma factors modulation [320] including ribosome and RNA polymerase engineering [321], can also be crucial for maximizing NPs output enhancing BGC expression. Finally, RNA-based post-transcriptional controls such as riboswitch [322] and riboregulators [323] offer additional tuning layers but remain underexploited for BGC regulation.

## Strategies in natural product analogues discovery

**Fig. 10:**
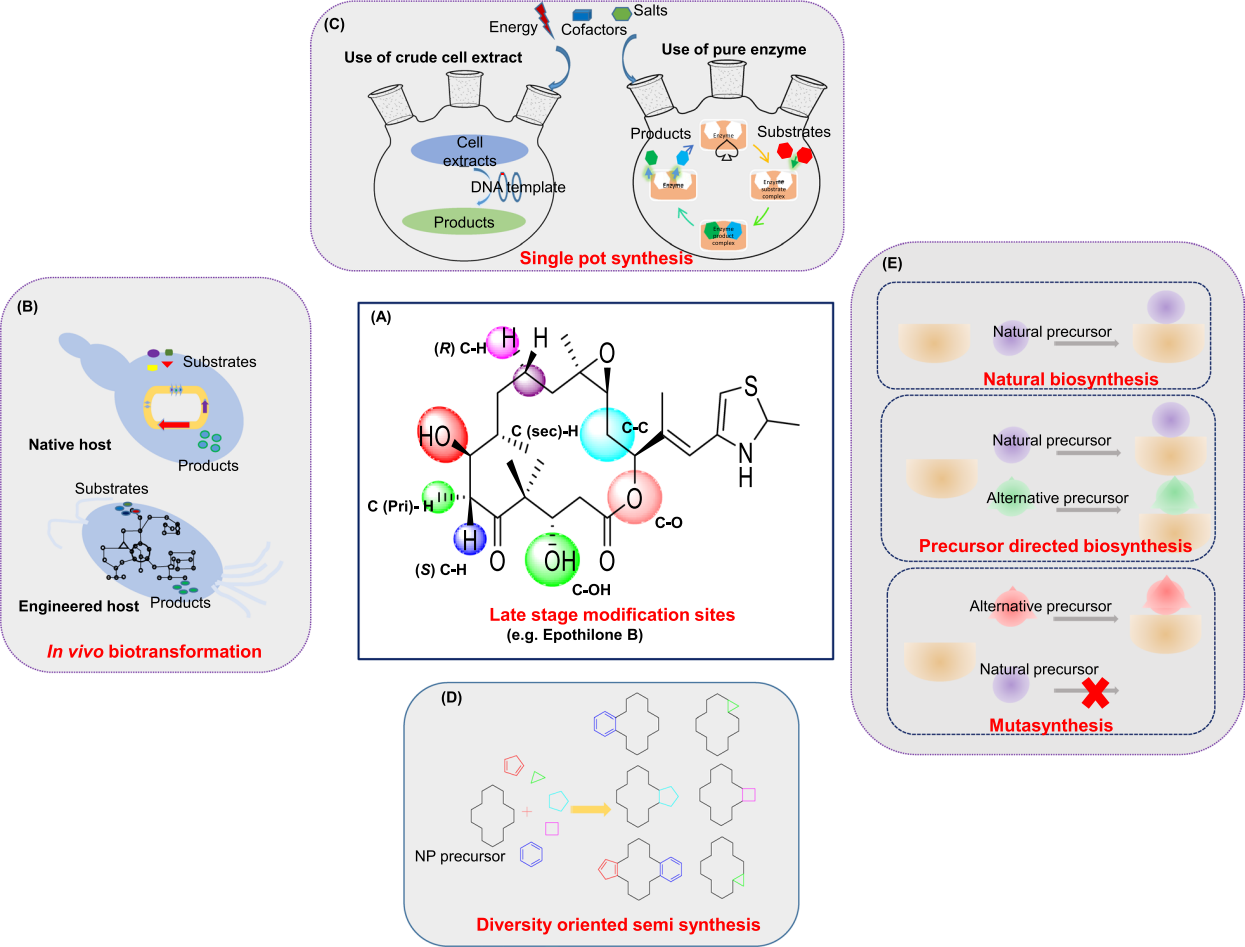
Schematic illustration of workflow of late stage diversification. **A** Late-stage functionalization in Epothilone B is illustrated through its molecular structure, where colored spheres highlight the specific bonds and functional groups modified. **B** In vivo biotransformation viz model organism and wild strain. **C** Single pot synthesis via cell free extract utilization and enzymatic recycle platform. **D** Diversity oriented semi synthesis. **E** Precursors directed and mutasynthesis

The abundance of stereo centers and unusual carbon skeletons (e.g. multiple- rings system, functional groups), along with the challenges of synthesis involving harsh reactions and critical (de) protection steps, pose significant challenges for NPs synthetic chemists to synthesize complex NPs scaffolds [324]. Discovery of novel catalysts, reagents, selective reactions, and strain selection, often combined with computational approaches, have enhanced NP diversification [325], even molecules not found in the nature. Efficient chemical modifications, like systematic ring-distortion and functional group additions (e.g. halogenation, oxidation, epoxidation), create NP-like small molecules with improved biological activities [324, 326]. Figure 10A highlight possible functionalizing of NP Epothilone B. Additionally, advances in large-scale metagenomics sequencing have uncovered novel biosynthetic enzymes with the ability to catalyze a wide array of chemical transformations [202], paving the way for microbial biotransformation and cell-free extract-based synthesis of valuable compounds or unprecedented chemical scaffolds.

### Late-stage diversification

Late-stage NP diversification through microbial biotransformation and cell-free approaches has gained interest for optimizing pharmacological properties and investigating structure–activity relationships [324].

#### Microbial biotransformation

Microbial biotransformation (Fig. 10B) employ either genetically engineered or wild strains to biosynthesize NP derivatives under mild conditions, avoiding complex purification steps low yields, and need for pure enzymes [327, 328] associated with chemical and biocatalyst [329]. Nevertheless, optimizing culture parameters (e.g. nutrient, pH, temperature) and selecting appropriate host strains [330] are utmost for maximizing productivity. These transformations effectively synthesize flavonoids [331], terpenes [332], glycosides, and vitamins analogues. For example, Huang et al. [331] employed recombinant *E. coli* whole cells to convert rutin into isoquercitrin, an antioxidant flavonoid glycoside. The process took place in a polyvinylidene fluoride membrane reactor that integrated reaction and separation, achieving over 80% conversion under mild conditions with efficient recyclability. Recent advances in microbial whole-cell biotransformation platforms, often integrated with semi-synthetic chemistry, feature multi-layered systems utilizing diverse wild type and engineered chassis strains. These strategies include co-cultivation of distinct microbial entities in both in vivo and in vitro setups to enhance biocatalytic efficiency [333]. Moreover, to streamline and improve scalability and productivity of NP analogues, attempts have also been made to implement continuous flow culture [334] or fed-batch culture [335], integrated of in vivo and in vitro platform [336], or micro droplets assisted synthesis [337].

In a recent study by Zhang et al. [333], developed hyper-porous hydrogel blocks for scalable cell encapsulation, ensuring nutrient access and limiting *E. coli* growth while supporting protein production. This enabled stable co-cultivation with *Streptomyces*, where encapsulated *E. coli* expressing RadH halogenase effectively halogenated genistein, unlike unencapsulated controls. Nevertheless, whole-cell biotransformation still faces key challenges, such as byproduct competition, toxicity of substrates, intermediates, and end product, and complex nutritional demands. The use of foreign enzymes can impose a metabolic burden, disrupting native pathways and affecting cell viability. Additionally, diverse nutritional needs and environmental conditions make co-culture systems challenging to maintain.

#### Cell-free platforms

As an alternative approach, cell-free platforms help to overcome the limitations of microbial biotransformation by enabling the NPs functionalization in a cell-free environment. Among various strategies within this framework, in vitro enzymatic methods [330] and diversity-oriented synthesis [338] have emerged as particularly effective, offering scalable and rapid NPs diversification. These methods leverage enzymes for stereo selective synthesis in a single pot, ideal for remote settings [339]. They handle toxic molecules and biosynthesize complex NPs like NRPS peptides, RIPPs peptides, cannabinoid, and limonene [340].

##### In-vitro enzymatic workflow

The NPs diversification using in vitro enzymatic cell-free workflows can be achieved through the use of purified enzymes [341], recombinant enzymes or crude cell extracts [342], alongside necessary cofactors, energy sources, and reactants, all integrated into a single “one-pot” reaction system as depicted in Fig. 10C. This strategy enables the biosynthesis of scaffold-specific NPs with high regio- and stereoselectivity. In particular, in vitro enzymatic cell-free workflows commonly employ redox tailoring enzymes, specialized biocatalysts that introduce oxidative modifications. Tailoring enzyme such as cytochrome P_450_ enzyme [343], *α*-ketoglutarate-dependent dioxygenases [344], flavin adenine dinucleotide or flavin mononucleotide- dependent oxygenases [345], play a pivotal role in diversifying primary and secondary metabolites through oxidative modifications, both *in-vitro* and *in-vivo*. For instance, the in vitro structural complexification of andiconin D was achieved using two α-ketoglutarate-dependent dioxygenases, SptF and SptN, which catalyzed oxidative transformations leading to the formation of the emervaridiones and emeridones[344]. Landscape of NP diversified through these methods have been described elsewhere [346, 347].

Nonetheless, it is not limited to tailoring enzymes alone; NP diversification also involves a wide array of other enzyme. For instance, Ditzel et al. [348] introduced a cell-free protein synthesis approach for constructing the NP caffeine. Their study highlighted how SAM-dependent methyltransferase reactions could be utilized within an in vitro framework to achieve partial biosynthesis. Specifically, the enzyme tea caffeine synthase was employed to catalyze caffeine production in this cell-free setup. A separate study reported the in vitro synthesis of indigoidine and rhabdopeptides using multidomain NRPSs, BpsA from *S.lavendulae* and KJ12ABC from *Xenorhabdus* KJ12.1, respectively [349].

##### Diversity oriented synthesis

Introduced in the early 2000s, diversity-oriented synthesis (DOS) emerged as a powerful approach for constructing structurally diverse molecular libraries for high-throughput screening. Specifically, it serves as a synthetic strategy for NP diversification, focusing on high structural complexity and molecular diversity while placing less emphasis on regio- and stereoselectivity [350, 351]. This approach enables chemists to retain the core NP scaffold while systematically modifying peripheral functionalities, stereochemical elements, or ring systems as illustrated in Fig. 10D. Remarkably, two key strategies are employed in this framework: the reagent-based approach, which modifies reagents or reaction conditions while keeping the substrate constant. For instance, use of amino acetophenones as building blocks for the synthesis of NP analogs such as 5- and 7-aminoflavones, azaflavones, 3-aryl-2-quinolones, epoxychalcones, azaaurones [352]. Other is substrate-based approach, which alters the starting substrate while keeping the reagents and conditions constant [353]. In particular, metals mediated reactions (e.g. Pd, Ni, Au, and Cu) [324] are commonly employed for selective NPs modification. Illustrative representative example of organocatlysis based diversity-oriented syntheses of 51 macrocycles with 48 unique scaffolds. By merging organocatalytic transformations with alkene metathesis, often in a one-pot setup, researchers achieved drug-like macrocycles with natural-product-like shape diversity [354].

Among several other strategic approaches, one is DOS libraries based on biosynthesis-inspired, as seen with PPAP analogues. These compounds are believed to derive from a desoxyhumulone core and two prenyl cation equivalents, which assemble the bicyclo [3.3.1] nonane scaffold via dearomative and alkene-intercepted prenylation. Leveraging this biosynthetic logic, a wide array of analogues with key bioactive structural features was synthesized [355]. In other cases, NPs-based hybrid molecules chemosynthesis, inspired by the unusual spiro-linked scaffold of indole–isatin hybrids, a DOS strategy was employed to synthesize 11 compounds, including dihydro- and tetrahydro-β-carbolines, piperidine- and pyrrolidine-fused β-carbolines, and spiropyrrolooxoindoles. Among them, two 1-aryltetrahydro-β-carbolines exhibited notable antimalarial activity [356]. Other multiple well-established DOS strategies for NP analogues discovery have been documented in the literature include diverted total synthesis, function-oriented synthesis, biology-oriented synthesis, and complexity-to-diversity. These approaches enhance chemical diversity and bioactivity, and are comprehensively reviewed elsewhere [357].

### Precursor-directed biosynthesis and mutasynthesis

Precursor-directed biosynthesis and mutasynthesis are versatile strategies combining biological and chemical methods to modify NPs. Precursor-directed biosynthesis uses altered or chemically synthesized precursors (Fig. 10E**)** in wild or modified strains to produce NP analogues. Examples include producing alkynyl- and alkenyl-substituted erythromycin A analogues in *E. coli* [358] and using isotope-labeled precursors for genomic studies, such as isolating novel polyketides from *P. fluorescens* [359]. This approach also aids in monitoring metabolic products using techniques like stable isotope probing Raman [360] and nano secondary ion MS (e.g. Nano SIMS) [361]. In a recent study by Zhang et al. [362] used single-pot, two-stage precursor-directed biosynthesis of diverse talaroenamines by *Penicillium malacosphaerulum* HPU-J01. In this approach, *p*-methylaniline initially served as a carrier to trap the biologically synthesized cyclohexanedione**,** forming talaroenamine F**.** Subsequently, various aniline derivatives were introduced to replace the p-methylaniline moiety, yielding the final products.

Challenges like competition between synthetic and natural precursors and poor intermediate incorporation rates can complicate purification and yields. These issues can be mitigated by blocking natural precursor synthesis through mutasynthesis [363], which involves mutating or inactivating key genes or adding specific enzyme inhibitors [358]. Improvement in omics, enzymology, bioinformatics, and tools like CRISPR-Cas have improved targeted gene knockouts, enhancing mutasynthesis. In particular, modern chassis strains like *Corynebacterium glutamicum, Dictyostelium discoideum, P. putida,* and *M. xanthus* are used for mutasynthesis-driven structural diversification. Successful story of this strategy include biosynthesis of halogenated actinomycin [364], pyrrole spiroketal [365], bipyridyl collismycin A [366], amychelin siderophores [367], and isopropylstilbene [368].

## Artificial intelligence in natural product discovery: innovations in screening, prediction, and metabolomics mining

**Fig. 11:**
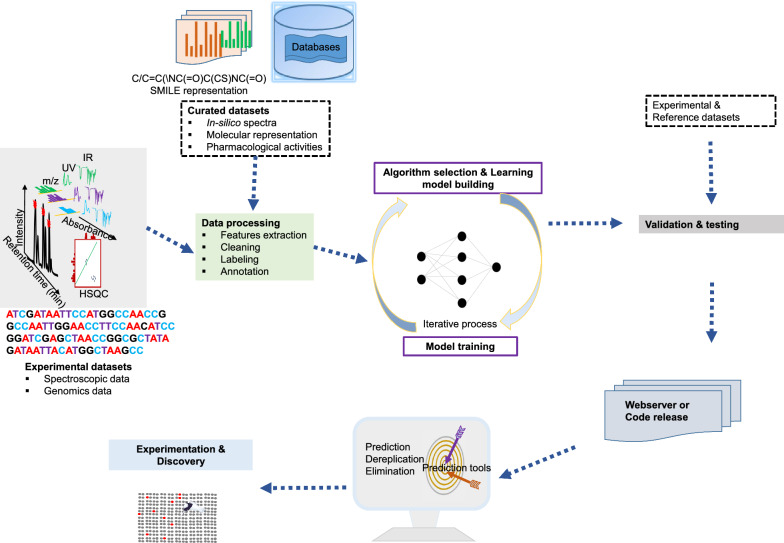
Overview of AI-Inspired advances in natural product discovery. The schematic illustrates the core stages involved in developing AI-driven automated tools for natural product research, utilizing either experimental spectroscopic data or curated datasets

**Fig. 12:**
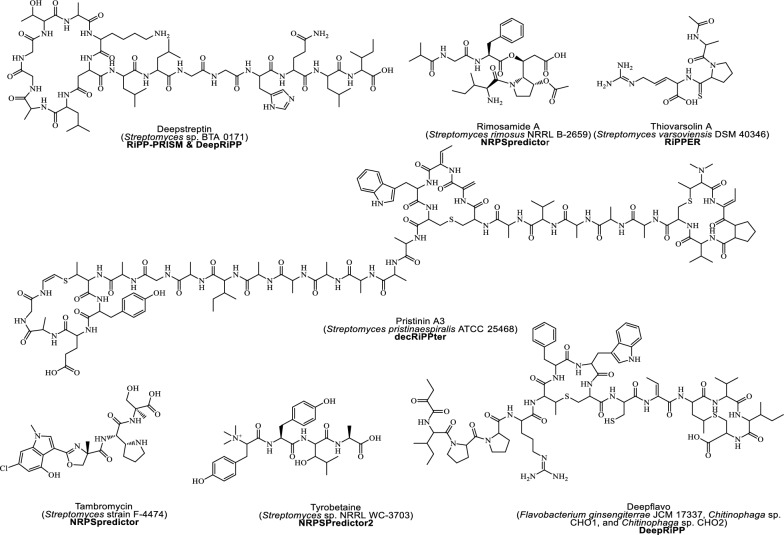
Molecular structures of selected drug candidates discovered using AI-powered techniques are shown, each labeled with its name, biological source (in parentheses), and the AI method applied (highlighted in bold)

This review has provided a concise discussion of NP strategies, yet a brief exploration of AI tools-driven discoveries in NPD would enhance its completeness. AI algorithms rapidly identify, categorize, and dereplicate compounds from complex mixtures, accelerating the discovery of novel NP, often with AI-powered web tools. The simplest variation among these tools relay on the choice of algorithms, quality of datasets for model generation, their accuracy of prediction or annotation accuracy, and computational efficiency [44]. AI has been applied across multiple facets areas of NP research, enhancing screening, pharmacological and molecular property prediction, and NP-inspired drug design. It also aids in NP target identification, deorphaning, genome and metabolomics mining, bio-/synthesis planning, classification, and structural characterization [369–371]. Among the current applications in NPD, most computational tools rely on building language models from simple rule-based systems to complex neural architectures that extract individual metabolite structures, chemical classes, or biosynthetic genes from experimental or archived datasets, such as genomic sequences or mass spectral profiles. Yet, given the structural and biosynthetic diversity of natural products, uniquely tailored models may be required to simultaneously predict multiple metabolite classes or reconstruct full biosynthetic pathways with higher fidelity [369]. NP-BGC identification largely relies on rule-based methods like AntiSMASH [29], PRISM [372] and CO-OCCUR [373], which effectively detect known BGCs but lacks in prediction of novel or unclustered pathways. Recent advances in machine learning and deep learning (DL) approaches have improved precision by recognizing unique BGC features. Tools like NPlinker [374], EFI-CGFP[375], and MAGI [376] aid biochemical enzyme or pathway predictions, while feature-based models such as BAGEL4 [377], SANDPUMA [378], and MetaMiner [379] classify metabolite types. ML-driven platforms like DeepBGC [223], GECCO [380], and SanntiS [381], outperform traditional methods in pattern recognition and scalability. Large-scale genome mining tools, BGC-Prophet [382], BiG-SLICE [39], BiG-SCAPE and CORASON [36]- enhance classification and phylogenetic analysis. Tools like decRiPPter [383], NeuRiPP [384] and DeepRiPP [385] have successfully predicted novel biosynthetic gene clusters, accelerating NP discovery. The BiG-FAM database, powered by ML, enhances gene cluster visualization and comparison [43]. ML-driven molecular networking classifier NPOmix [386] and DeepSAT [387] further refines novel NP discovery by refining MS and NMR analysis, respectively, addressing spectral reconstruction and molecular annotation challenges. Modern DL models (e.g. BiGCARP) [388], capable of predicting biosynthetic routes from NP structures, offer a foundation for matching NP to their respective BGCs. Currently landscape studies have focused on AI-guided NPD enabling the NP discovery. We emphasized these reviews to provide the reader with a deeper understanding of the subject [44, 369–371]. Additional, AI tools employed in NPD are presented in Table 1B–C, along with their respective descriptions. Furthermore, Fig. 11 presents a schematic workflow for the design of AI-inspired prediction tools in NPD, while Fig. 12 showcases the unique metabolite structures that have been mined from natural sources through AI-driven approaches.

## Conclusions

NPs offer holistic benefits for human health and sustainability, with advantages over synthetic compounds such as diverse chemical scaffolds, strong biocompatibility, and eco-friendliness. However, their low titers under standard conditions often fall below levels required for isolation and characterization, making scalable production challenging. Recent advances in analytical tools, dereplication, and genomic techniques have improved NPs targeting, discovery and identification, while BGCs refactoring and metabolic engineering have enabled limited success in boosting and diversifying NPs yields. Nevertheless, discovery remains slow, labor-intensive, and prone to rediscovery of known compounds. Downstream processing complexity, high costs, and infrastructure demands further limit the adoption of advanced methods, leaving many labs reliant on traditional approaches. Therefore, to enable characterization-scale production of novel and complex NPs, more rational and efficient strategies for improving titers are urgently needed. Although modern sequencing and bioinformatics reveal rich biosynthetic potential in microbial genomes, less than 3% of BGCs have been experimentally characterized [389]. Unlocking silent or poorly expressed BGCs remains a major challenge due to the inherent complexities of NP research, as summarized in Table 3.

To overcome this bottleneck, next-generation automatic tools, including brain-computer interfaces and robotics, are advisable, and should focus on high-resolution chromatographic techniques, integrated omics approaches, and automated metabolomics platforms including, (1) Developing tools or protocols to activate majority of silent biosynthetic gene clusters and selecting efficient elicitors or optimization of culture conditions. (2) Developing integrated methods to isolate, sequence, and sort specific microbial strains from complex environmental samples. (3) Designing fully automated metabolomics platforms to streamline extraction, purification, structural elucidation, bioassays, and prediction of putative biological targets. (4) Creating efficient and affordable biosynthetic systems. (5) Implementing self-optimizing techniques for NPs functionalization and characterization. (6) Developing tools that correlate chemical spectral data with previously uncharacterized BGCs. Lastly, serendipity, continuous efforts, and patience remain crucial for groundbreaking discoveries and advancements in NP research.

## Data Availability

No primary research results, software or codes have been included and no new data were generated or analysed as part of this review and examples of suitable statements you can use.
